# The intestinal microbiota and metabolites in patients with anorexia nervosa

**DOI:** 10.1080/19490976.2021.1902771

**Published:** 2021-03-28

**Authors:** Petra Prochazkova, Radka Roubalova, Jiri Dvorak, Jakub Kreisinger, Martin Hill, Helena Tlaskalova-Hogenova, Petra Tomasova, Helena Pelantova, Martina Cermakova, Marek Kuzma, Josef Bulant, Martin Bilej, Kvido Smitka, Alena Lambertova, Petra Holanova, Hana Papezova

**Affiliations:** a Laboratory of Cellular and Molecular Immunology, Institute of Microbiology of the Czech Academy of Sciences, Prague, Czech Republic; b Faculty of Science, Department of Zoology, Charles University, Prague, Czech Republic; c Department of Steroids and Proteohormones, Institute of Endocrinology, Prague, Czech Republic; d Laboratory of Molecular Structure Characterization, Institute of Microbiology of the Czech Academy of Sciences, Prague, Czech Republic; e 4th Medical Department, First Faculty of Medicine, Charles University and General Faculty Hospital, Prague, Czech Republic; f Department of Psychiatry, First Faculty of Medicine, Charles University and General University Hospital in Prague, Prague, Czech Republic; g Department of Paediatrics and Inherited Metabolic Disorders, First Faculty of Medicine, Charles University and General University Hospital in Prague, Prague, Czech Republic; h First Faculty of Medicine, Institute of Physiology, Charles University, Prague, Czech Republic; i First Faculty of Medicine, Institute of Pathological Physiology, Charles University, Prague, Czech Republic

**Keywords:** Microbiome, bacteriome, mycobiome, SCFA, neurotransmitter, EDE-Q, BMI, dysbiosis, renourishment, gut-brain-microbiota axis

## Abstract

Brain-gut microbiota interactions are intensively studied in connection with various neurological and psychiatric diseases. While anorexia nervosa (AN) pathophysiology is not entirely clear, it is presumably linked to microbiome dysbiosis. We aimed to elucidate the gut microbiota contribution in AN disease pathophysiology. We analyzed the composition and diversity of the gut microbiome of patients with AN (bacteriome and mycobiome) from stool samples before and after renourishment, and compared them to healthy controls. Further, levels of assorted neurotransmitters and short-chain fatty acids (SCFA) were analyzed in stool samples by MS and NMR, respectively. Biochemical, anthropometric, and psychometric profiles were assessed. The bacterial alpha-diversity parameter analyses revealed only increased Chao 1 index in patients with AN before the realimentation, reflecting their interindividual variation. Subsequently, core microbiota depletion signs were observed in patients with AN. Overrepresented OTUs (operation taxonomic units) in patients with AN taxonomically belonged to *Alistipes, Clostridiales, Christensenellaceae*, and *Ruminococcaceae*. Underrepresented OTUs in patients with AN were *Faecalibacterium, Agathobacter, Bacteroides, Blautia*, and *Lachnospira*. Patients exhibited greater interindividual variation in the gut bacteriome, as well as in metagenome content compared to controls, suggesting altered bacteriome functions. Patients had decreased levels of serotonin, GABA, dopamine, butyrate, and acetate in their stool samples compared to controls. Mycobiome analysis did not reveal significant differences in alpha diversity and fungal profile composition between patients with AN and healthy controls, nor any correlation of the fungal composition with the bacterial profile. Our results show the changed profile of the gut microbiome and its metabolites in patients with severe AN. Although therapeutic partial renourishment led to increased body mass index and improved psychometric parameters, SCFA, and neurotransmitter profiles, as well as microbial community compositions, did not change substantially during the hospitalization period, which can be potentially caused by only partial weight recovery.

## Introduction

Anorexia nervosa (AN) is a complex eating disorder characterized by self-starvation, excessive weight loss, modified body self-perception, and an intense fear of gaining weight. This severe psychiatric illness is one of the most common chronic diseases with onset in female adolescence, usually gradually associated with various medical and psychiatric comorbidities. AN has the greatest mortality rates of any psychiatric disorder in young females. According to the criteria of the Diagnostic and Statistical Manual of Mental Disorders (DSM-5), patients with AN can be classified as restrictive or binge-eating/purging subtypes. While the restrictive subtype is characterized by starvation and frequent physical hyperactivity, the binge-eating/purging subtype is defined by self-induced vomiting and misusing laxatives, diuretics, or enemas.^1^

The study of the human microbiome, including bacteria, archaea, fungi, viruses, and protozoa, in physiology regulation in both health and disease is recently widely extending. Many diseases, ranging from autoimmune, neurodegenerative, or even cancers, are linked to microbiome imbalance, called dysbiosis.^2^ Microbiome dysbiosis is characterized by either expansion of pathobionts, loss of commensals, loss of microbial diversity, or their combinations.^3^ The main focus is dedicated to the bacterial microbiome portion. About 1,000 bacterial species colonize the human gut, and two-thirds of them are unique to each individual.^4^ Non-negligible constituents of human microbiota are fungi, representing the so-called mycobiome. Fungi represent only 0.001–0.1% of genes found in gut microbiome stool samples, and their diversity is much lesser compared to bacteria. Further, the mycobiome exerts great inter- and intra-individual variability, and there is no consensus on the normal balanced fungal community composition.^5^ Microbiota composition and its associated metabolites can be influenced by many factors such as diet, hygiene, geographical location, host genotype, age, medicaments, etc.^6^ Diet and starvation as well as anxiety and stress, which are often associated with AN disorder, can alter the patient’s microbiome.^7^

Currently, there are a few studies on the gut microbiota in patients with AN; all performed on stool samples. The outcomes of these studies are diverse and deviations in abundance, diversity, and microbial composition were found. The number of patients with AN in cross-sectional studies was mostly few, between 9 and 25.^8–12^ Two longitudinal studies included 16 and 55 patients.^7,13^ Lesser alpha diversity at least in one analyzed index, reflecting the variance within a particular sample compared to controls, was described in four studies;^7,12,14,15^ in two other studies, no difference in diversity was found.^11,13^ Concerning specific changes in microbiota composition, there are some indications of different signatures associated with AN. One potentially relevant species is the archaeon *Methanobrevibacter smithii*, which abundance was increased in several AN studies.^8,10,11,13^ Conversely, decreased species abundance from the phylum Firmicutes, e.g. *Roseburia, Clostridium, Anaerostipes, and Faecalibacterium*, was reported as a significant feature of patients with AN.^7,11,13,14^

The central nervous system (CNS) and the intestine are closely connected. The gut and the host brain bidirectionally communicate, and thus represent the so-called gut-brain axis. CNS modulation by the microbiome occurs primarily through neuroimmune and neuroendocrine mechanisms. This communication is mostly mediated by gut microbial metabolites, including short-chain fatty acids (SCFAs), bile acids, tryptophan metabolites, and various neurotransmitters and hormones.^16,17^ Specifically, microbes were shown to synthesize dopamine, serotonin, norepinephrine, and gamma-aminobutyric acid (GABA).^16^ SCFAs are generated by microbial fermentation of non-digestible colon polysaccharides. The majority of gut microbial-derived SCFAs represent acetate, propionate, and butyrate.^18^ Produced microbial metabolites may enter systemic circulation, cross the blood-brain barrier, affect brain structures, and thus modify various cognitive functions.^19,20^

We tested the hypothesis that gut microbiota and its metabolites in patients with AN differ from healthy controls. We aimed to identify hallmarks of AN microbiota, to assess their changes during realimentation, to determine the levels of assorted neurohormones and SCFAs at hospitalization admission and discharge, and to identify potential correlations with various biochemical as well as anthropometric and psychometric parameters. While previous intestinal microbiota studies mainly focused on bacterial species, other microbiota members, e.g. fungi, archaea, viruses, and protozoa, are also relevant in microbiome analysis. In this study, fungal community composition was also assessed. Overall, this study represents a detailed longitudinal study of 52 patients with AN.

## Results

### Parameters of healthy women and patients with AN prior to (AN1) and after hospitalization (AN2)

The basic parameters of all studied groups (control, AN1, AN2) are summarized (Table 1). Patients with AN (AN1) differed from healthy women significantly in body mass index (BMI), body fat percentage, and waistline and hipline circumference. Further, they had significantly decreased levels of total protein, alpha 1 globulin, beta globulin, gamma globulin, IgG, IgM, cholinesterase, and fT4. Conversely, they exhibited increased levels of albumin percentage (not albumin concentration) and IL-17 (Table 1). The alteration of these biochemical blood parameters is related to malnutrition and protein deficiency from food.

**Table 1.** Initial and final parameter values of controls and patients with AN

**Variable**	**Control**	**AN1**	**AN2**	**∆** **(AN2-AN1)**	**p-value** **for ∆**	**p-value**	**p-value**	**p-value**	**p-value** **for Kruskal-Wallis test**	**Normal adult womenreference ranges**
						**AN1 vs. AN2**	**AN1 vs. C**	**AN2 vs. C**		
Age (years)	24 (22, 28.5)	23 (19, 27)								
Hospitalization (days)			51 (38.5, 64)							
Disease duration (months)		60 (36, 126)								
Height (cm)	169 (167, 173)	165 (162, 170)								
BMI (kg/m^2^)	21.9 (19.9, 23.7)	14.4 (13.4, 15.9)	17.1 (15.5, 18.1)	2.18 (1.53, 3.2)	<0.001	***	***	***	<0.001	
Body Fat (%)	24.2 (21.1, 28.7)	3 (3, 7.2)	9 (3.43, 15.2)	3.4 (0, 7)	<0.001	*	***	***	<0.001	
Waist (cm)	70 (66, 74)	57.5 (55, 61)	64 (61, 66.8)	6 (4, 8)	<0.001	***	***	***	<0.001	
Hip (cm)	94 (90.5, 100)	77 (74, 80)	83 (79.3, 84.8)	4 (3, 7)	<0.001	**	***	***	<0.001	
Total protein (g/l)	69.2 (66.7, 71.5)	66.7 (63.6, 70.5)	67.6 (64.6, 69.9)	0.2 (-3.65, 3.23)	NS		**	*	<0.05	65-85
Albumin (%)	57.5 (55.4, 59.8)	60.2 (58.7, 62.8)	58.5 (57, 60.3)	-2.4 (-4.3, -0.675)	<0.001	**	***		<0.001	55-69
Albumin (g/l)	40.4 (38.2, 41.7)	40.5 (38.1, 42.2)	39.2 (38.1, 40.5)	-1.55 (-3.08, 0.325)	NS				NS	-
α1 globulin (%)	2.5 (2.2, 2.8)	2.2 (1.9, 2.5)	2.2 (2, 2.7)	0 (-0.2, 0.2)	NS		**		NS	1.5-4
α1 globulin (g/l)	1.7 (1.55, 1.9)	1.5 (1.3, 1.7)	1.5 (1.35, 1.8)	0 (-0.2, 0.2)	NS		***	**	<0.01	-
α2 globulin (%)	11.9 (10.9, 12.9)	11.9 (10.9, 12.3)	12.4 (11.9, 13.3)	0.8 (0.5, 1.63)	<0.001	**		*	NS	8-12
α2 globulin (g/l)	8.1 (7.45, 8.95)	7.8 (7.05, 8.4)	8.4 (7.8, 9)	0.6 (0.2, 1.4)	<0.001	**			NS	-
β globulin (%)	11.9 (11, 13)	11 (10.2, 11.9)	12.4 (11.3, 13)	1.4 (0.975, 2.13)	<0.001	***	***		<0.001	7-15
β globulin (g/l)	8.4 (7.7, 9.1)	7.3 (6.6, 8.1)	8.3 (7.6, 9)	0.8 (0.3, 1.9)	<0.001	***	***		<0.001	-
γ globulin (%)	16 (14.2, 17.7)	14.1 (12.2, 16.2)	14 (12.6, 16.3)	0 (-0.95, 0.925)	NS		**	**	<0.05	9-18
γ globulin (g/l)	11.1 (9.65, 12.7)	9.6 (7.7, 11.4)	9.7 (8.15, 11.1)	0.2 (-0.625, 0.875)	NS		***	**	<0.01	-
IgG (g/l)	11.2 (9.94, 12.6)	9.97 (8.2, 12)	10.5 (9.01, 12.3)	0.185 (-0.305, 0.88)	NS		*		NS	6.7-15
IgA (g/l)	1.72 (1.32, 2.27)	1.93 (1.53, 2.33)	1.9 (1.37, 2.2)	-0.13 (-0.265, 0.0125)	<0.01				NS	0.9-3.7
IgM (g/l)	1.36 (1.15, 1.68)	1.03 (0.76, 1.41)	1.05 (0.68, 1.27)	-0.005 (-0.085, 0.0425)	NS		***	***	<0.001	0.6-2.2
IgE (IU/ml)	36.6 (12.8, 86.4)	23.9 (9.65, 77.4)	18.4 (8.8, 75.1)	-0.75 (-5.78, 0.325)	NS				NS	0-200
CRP (mg/l)	0.76 (0.34, 1.7)	0.61 (0.36, 0.89)	0.56 (0.18, 1.04)	0.0219 (-0.398, 0.511)	NS				NS	2-8
Cholinesterase (ukat/l)	108 (100, 123)	93 (77.5, 111)	104 (95.5, 118)	14 (4, 23.5)	<0.001	*	***		<0.01	85.2-195.4
TAG (mmol/l)	0.85 (0.59, 1.08)	0.85 (0.59, 1.02)	0.92 (0.68, 1.2)	0.07 (-0.06, 0.333)	NS				NS	0.45-1.7
TSH (mIU/L)	2.36 (1.92, 3.44)	2.11 (1.43, 2.7)	2.3 (1.78, 3.41)	0.3 (-0.423, 0.749)	NS				NS	0.3-3.5
fT4 (pmol/l)	14.8 (13.8, 15.8)	12.5 (11.9, 13.6)	12 (10.7, 12.9)	-0.6 (-1.93, 0.5)	NS		***	***	<0.001	12-22
IL-6 (pg/ml)	1.51 (1.14, 2.15)	1.6 (0.985, 2.76)	1.65 (1.01, 2.22)	0.06 (-0.53, 1.01)	NS				NS	-
TNF-α (pg/ml)	1.02 (0.91, 1.17)	1.09 (0.88, 1.33)	1.11 (0.95, 1.28)	0.08 (-0.065, 0.183)	NS				NS	-
IL-17 (pg/ml)	0 (0, 0.28)	0.34 (0.03, 1.21)	0.52 (0.16, 0.9)	0.055 (-0.123, 0.473)	NS		***	**	<0.001	-

The multiple comparisons between groups were evaluated by the Kruskal-Wallis Z test followed Dunn’s multiple comparisons with Bonferonni correction. Changes during hospitalization (calculated as the values at hospitalization end and beginning) were evaluated by Wilcoxonʼs paired test corrected for ties. Both Kruskal-Wallis test and Wilcoxon’s test were corrected for multiplicity using Bonferroni correction. The results are shown as median with quartiles. AN1 – patients with anorexia nervosa before treatment, AN2 –patients with anorexia nervosa after treatment; ∆ represents the absolute change calculated as the value after intervention - basal value; TAG – triacylglyceride; TSH – thyroid-stimulating hormone; fT4 – free thyroxine; TNF-α – tumor necrosis factor; IL – interleukin. nControl=67, nAN1=52, nAN2=52; *p<0.05, **p<0.01, ***p<0.001. NS – non-significant.

Patient renourishment during hospitalization led to increased BMI, body fat percentage, waist and hip circumference, alpha 2 and beta globulin and cholinesterase levels, which were observed at their discharge (∆ values), whereas albumin and IgA levels were decreased (Table 1). However, some patients’ values at their discharge did not reach control values, suggesting that patients with AN were not fully recovered.

### Eating disorder examination-questionnaire (EDE-Q)

The mean EDE-Q global and subscale scores of patients with AN and their changes during treatment are shown in Table 2. All EDE-Q concern values considerably decreased after treatment, indicating eating disorder psychopathology improvement (Table 2). All four categories, as well as a total score, correlate with each other having the greatest levels in total score (Table S1).

**Table 2.** Initial and final EDE-Q scores and their changes

Variable	AN1	AN2	∆	p-value	
EDE-Q restraint concern	2.4 (0.9, 4.3)	0.4 (0.05, 0.8)	−1.8 (−3.6, −0.6)	˂0.001	***
EDE-Q eating concern	3 (1.6, 4)	1 (0.4, 1.4)	−1.6 (−2.5, −0.5)	˂0.001	***
EDE-Q shape concern	3.88 (2.88, 5.13)	2.88 (2.03, 4.22)	−0.625 (−1.88, 0.125)	˂0.001	***
EDE- Q weight concern	3.4 (2.2, 4.8)	1.8 (1, 3)	−1 (−2, −0.2)	˂0.001	***
EDE-Q total score	3 (1.95, 4.46)	1.37 (0.988, 2.63)	−1.21 (−2.32, −0.563)	˂0.001	***

∆ represents the absolute change calculated as the value after intervention – basal value. The *p*-value was determined by Wilcoxon’s robust paired test; AN1 – patients with anorexia nervosa before treatment, AN2 – patients with anorexia nervosa after treatment, nAN1 = 59, nAN2 = 52. Standardized Cronbach’s alpha coefficient was = 0.95 for EDE-Q scores and 0.91 for EDE-Q score changes.

### Body mass index (BMI)

At patients’ admission, lesser levels of total protein, alpha 1 globulin, beta globulin, gamma globulin, IgG, and cholinesterase correlated with BMI values (data not shown). Orthogonal projections to latent structures (OPLS) analysis indicated that the increase in BMI during realimentation correlated positively with hospitalization length and negatively with adulthood stress. Further, the BMI increase correlated with the decrease in Eating Disorder Examination Questionnaire (EDE-Q) components, fT4 levels, and AN severity, and with an increase in gamma globulin and IgM levels. The multiple regression (MR) model of the same data revealed that some parameters did not reach significance; thus, they are not independent and they are intercorrelated with other explaining variables (Table 3). The variables in the OPLS model and MR analysis explained 80.2% (69.3% after cross-validation) of the variability in the BMI changes.

**Table 3.** Relationships between ∆BMI and relevant parameters as evaluated by the OPLS model and multiple regression analyses

		OPLS				Multiple regression			
	Variable	Component loading	t-statistics	R*^a^*		Regression coefficient	t-statistics		
Relevant predictors (matrix **X**)	∆EDE-Q eating concern	−0.297	−3.04	−0.469	**	−0.089	−1.73		
	∆EDE-Q weight concern	−0.23	−2.41	−0.364	*	0.023	0.3		
	Hospitalization (days)	0.525	13.79	0.83	**	0.372	3.83	**	
	Adulthood stress	−0.285	−8.05	−0.451	**	−0.131	−1.89		
	∆Gamma globulin (%)	0.257	1.97	0.406	*	0.078	1.25		
	∆Gamma globulin (g/l)	0.241	2.26	0.381	*	0.088	1.63		
	∆IgM (g/l)	0.226	2.66	0.357	*	0.15	2.43	*	
	∆fT4 (pmol/l)	−0.217	−2.53	−0.343	*	−0.167	−2.09	*	
	∆AN DSM mild	0.315	2.88	0.498	*	0.289	2.78	*	
	∆AN DSM extreme	−0.324	−4.74	−0.513	**	−0.225	−3.9	**	
(matrix **Y**)	∆BMI (kg/m^2^)	1	20.95	0.896	**				
**Explained variability**		80.2% (69.3% after cross-validation)							

^a^R Component loadings expressed as correlation coefficients with predictive component, *p < 0.05, **p < 0.01; fT4 – free thyroxine

BMI change in patients with AN depends on many parameters, and it can be to a certain extent predicted. Prediction analysis (OPLS) revealed that reduced BMI, body fat percentage, smaller hipline, adulthood stress, and basic education worsen the conditions for BMI increase (Table 4). MR analysis showed a similar significance pattern; only the hipline value seems to be dependent on other predictors (Table 4). It is likely that lesser BMI, body fat percentage, and hipline predict greater BMI increase due to the greater distance to normal values. The predictors in the OPLS model and MR analysis explained 53.5% (44.4% after cross-validation) of the variability in the BMI changes.

**Table 4.** Prediction of ∆BMI and predictors as evaluated by the OPLS model and MR

		OPLS					Multiple regression		
	Variable	Component loading		t-statistics	R*^a^*		Regression coefficient	t-statistics	
Relevant predictors (matrix **X**)	BMI (kg/m^2^)		−0.365	−4.354	−0.471	**	−0.237	−3.45	**
	Body Fat (%)		−0.291	−3.54	−0.378	**	−0.184	−4.44	**
	Hip (cm)		−0.323	−2.79	−0.419	*	−0.124	−1	
	Adulthood stress		−0.501	−6.03	−0.659	**	−0.202	−2.29	*
	Basic education		−0.432	−3.19	−0.54	**	−0.428	−4.71	**
(matrix **Y**)	∆BMI (kg/m^2^)	1		9.77	0.732	**			
**Explained variability**		53.5% (44.4% after a cross-validation)							

^a^R Component loadings expressed as correlation coefficients with predictive component, *p < 0.05, **p < 0.01

### Outcome predictors

Prediction analysis (OPLS) revealed that shorter hospitalization duration, longer illness duration, lesser BMI, lesser total protein and albumin concentrations, adulthood stress, comorbid somatic diagnosis associated with antidepressant and other medication, and disability pension result in worse patient outcome (Table 5). But OPLS analysis did not find the significance of kynurenine, acetate (described in the subsequent metabolite section), and IgE levels. The OPLS model and MR analysis predictors explained 46.4% (35.7% after cross-validation) of the outcome variability. The associations between the negative outcome as an explained variable and various parameters analyzed by OPLS and multiple regression revealed similar results. The OPLS model and MR analysis predictors explained 53.9% (47.9% after cross-validation) of the outcome variability (Table S2).

**Table 5.** Prediction of patients’ outcome and predictors as evaluated by the OPLS model and MR

		Predictive component					Multiple regression		
	Variable	Component loading	t-statistics	R*^a^*			Regression coefficient	t-statistics	
Relevant predictors (matrix **X**)	Hospitalization duration (days)	−0.185	−1.90	−0.328	*		−0.114	−2.60	*
	Disease duration (months)	0.338	7.94	0.596	**		0.110	3.41	**
	BMI (kg/m^2^)	−0.167	−2.54	−0.294	*		−0.055	−2.53	*
	Total protein (g/l)	−0.209	−4.22	−0.369	**		−0.069	−1.62	
	Albumin (g/l)	−0.207	−3.13	−0.367	**		−0.075	−2.13	*
	IgE (IU/ml)	−0.003	−0.05	−0.007			−0.058	−2.25	*
	Kynurenine (PQN)	−0.146	−1.27	−0.253			−0.093	−2.93	*
	Acetate (PQN)	0.171	1.81	0.290			0.065	2.74	*
	Adulthood stress	0.252	2.77	0.447	*		0.110	2.60	*
	No somatic diagnosis	−0.373	−7.64	−0.657	**		−0.119	−2.60	*
	Disability pension	0.395	5.64	0.698	**		0.146	2.95	*
	University education	−0.061	−0.97	−0.107			−0.063	−2.57	*
	Second education	−0.135	−2.29	−0.238	*		−0.054	−3.81	**
	Basic education	−0.011	−0.27	−0.020			−0.039	−1.94	*
	No medication	−0.382	−5.93	−0.673	**		−0.100	−4.31	**
	Antidepressants	0.261	3.65	0.461	**		0.096	2.04	*
	Other medication	0.429	8.93	0.758	**		0.137	3.81	**
(matrix **Y**)	Negative outcome, LRR^b^	1.000	6.73	0.681	**				
**Explained variability**		46.4% (35.7% after cross-validation)							

^a^R Component loadings expressed as correlation coefficients with predictive component, *p < 0.05, **p < 0.01

^b^LRR Logarithm of likelihood ratio (logarithm of the ratio of the probability that the patient’s psychopathology improved to the probability that not); PQN–probabilistic quotient normalization

### Gut bacteriome

DNA isolation processing quality as well as amplicon libraries preparation workflow were demonstrated by microbial community standards sequencing (Table S3). The relative bacterial abundance approximately corresponded to the original standards composition except for *Pseudomonas aeruginosa* abundance, which was present in the analyzed sample in a greater proportion than expected, particularly in a microbial community with log distribution of bacterial species. However, the standards provide only theoretical composition calculated from theoretical genomic DNA composition, taking into account the genome size and 16S copy number.

The bacterial dataset comprised 997 operation taxonomic units (OTUs; including six archaeal OTUs) represented by 5,649,978 high-quality sequences with average sequencing depth per sample corresponding to 32,849 reads (range: 6723–117,647). Both observed OTUs number and estimated Chao 1-based total OTU richness varied between the studied groups (control, AN1, AN2; LMM: Δ d.f. = 2, χ^2^ = 7.0287, *p* = 0.02977; Δ d.f. = 2, χ^2^ = 7.8437, *p* = 0.0198, respectively). According to Tukey post-hoc testing, the Chao 1 index was increased in AN1 compared to controls (*p* = 0.0384), and compared to AN2 (*p* = 0.0482). The same marginally nonsignificant trend was observed if analyzing observed OTU richness (Tukey post-hoc tests: *p* = 0.0588 for AN1 vs. control groups and *p* = 0.0596 for AN1 vs. AN2). However, there was no significant difference in observed Shannon diversity between the studied groups (LMM: Δ d.f. = 2, χ^2^ = 3.1526, *p* = 0.2067, Figure 1).

**Figure 1.**
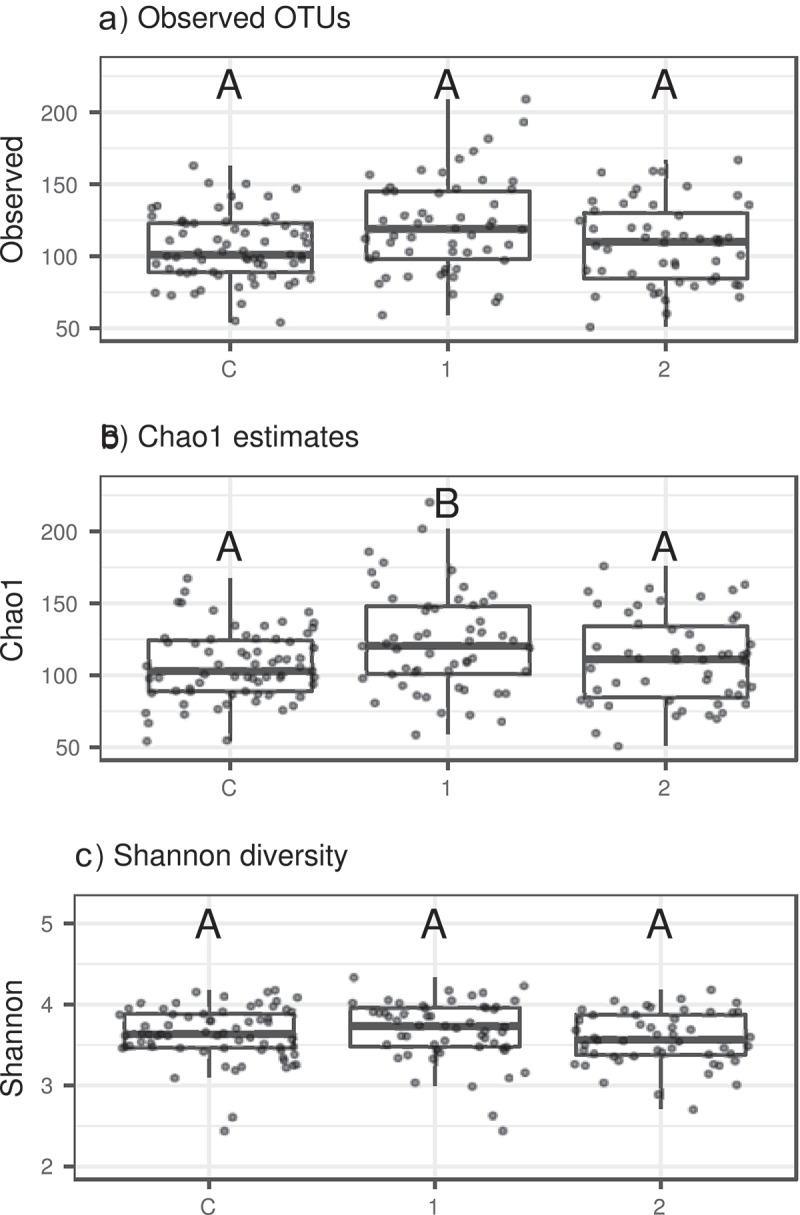
Gut bacteriome alpha diversity variation between control individuals vs. AN1 vs. AN2 assessed based on A) Observed OTUs number, B) Total OTU richness predicted by Chao 1 index, and C) Shannon index. Significant differences between categories (*p* < 0.05 according to Tukey post-hoc tests) are indicated by different letters above the bars

**Figure 2.**
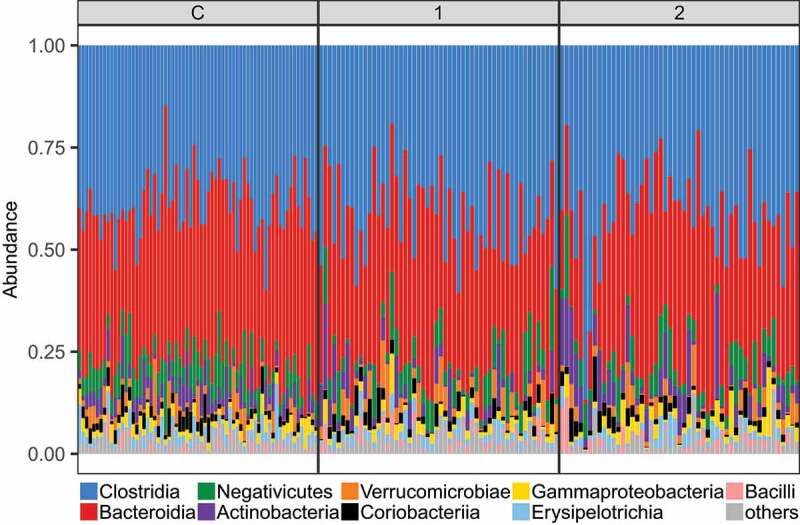
Proportions of dominating bacterial classes (represented by >1% of reads) in the three studied groups

Bacterial profiles of the three studied groups exhibited comparable representation of dominating bacterial classes (Figure 2). Similarly, we did not observe any pronounced differences in average proportions of bacterial genera between the three groups (Fig. S1).

**Figure 3.**
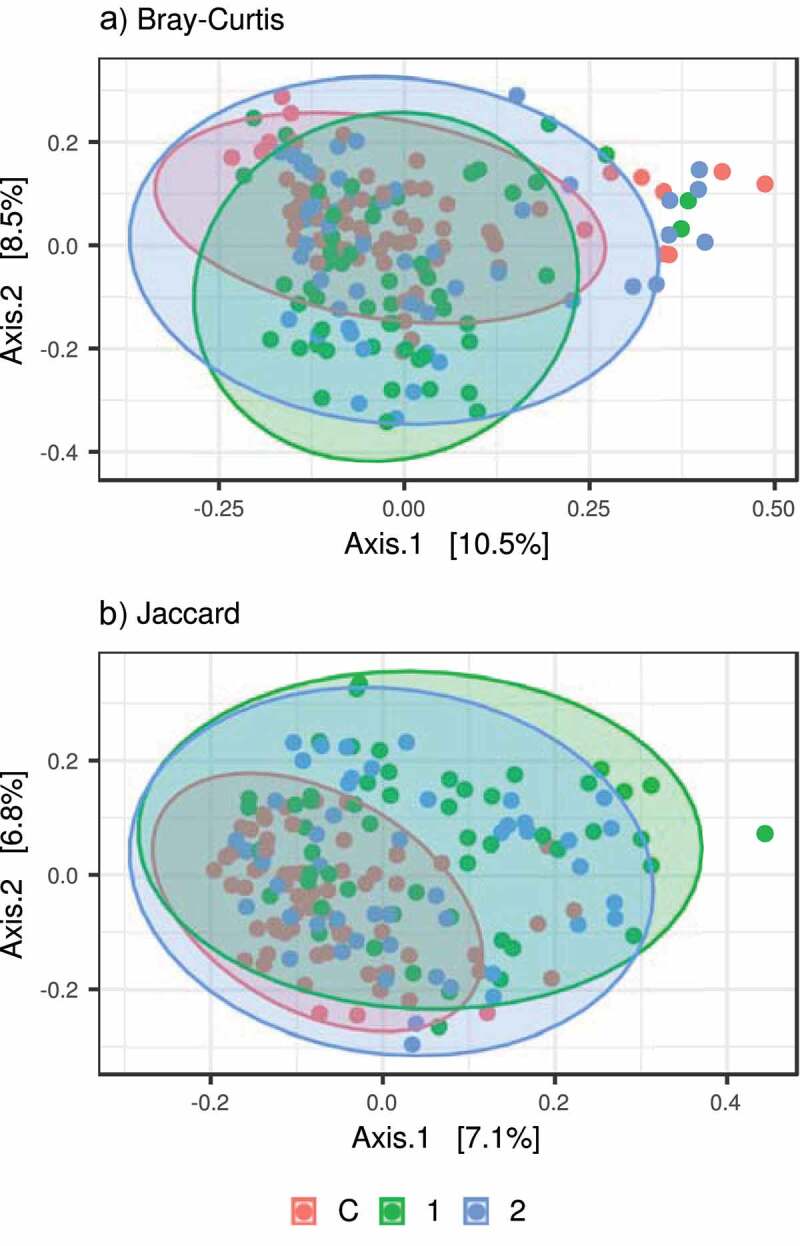
PCoA showing variation in bacterial microbiota composition between controls vs. AN1 vs. AN2. Compositional variation was assessed based on A) relative abundance-based (Bray-Curtis) and B) prevalence-based (Jaccard) dissimilarities

According to betadisper analysis, the control group exhibited reduced interindividual gut bacteriome variation compared to both patient groups, whereas the interindividual variation of AN1 did not differ from AN2 (Table 6). Furthermore, pair-wise PERMANOVA analyses suggested systematic differences in gut bacteriome composition between control samples and both patient groups, but not between AN1 and AN2 (Table 6 and Figure 3). However, interindividual variation and bacterial composition changed partially during the therapy as can be seen from pair-wise comparison values (Table 6). Overall, the microbiome of patients with AN after weight gain more resembles the microbiome of patients with AN before renourishment than that of healthy controls.

**Table 6.** Pair-wise comparisons of gut bacteriome interindividual variation and composition

			Bray-Curtis			Jaccard		
**A) BETADISPER**		**df**	**F**	**p**		**F**	**p**	
	control vs. AN1	1;119	**31.597**	**0.001**		**33.928**	**0.001**	
	control vs. AN2	1;116	**16.492**	**0.001**		**20.316**	**0.001**	
	AN1 vs. AN2	1;103	1.258	0.280		1.539	0.217	
**B) PERMANOVA**		**df**	**F**	**p**	**R^2^**	**F**	**p**	**R^2^**
	control vs. AN1	1;119	**3.595**	**0.001**	0.029	**4.013**	**0.001**	0.033
	control vs. AN2	1;116	**2.309**	**0.001**	0.020	**2.370**	**0.001**	0.020
	AN1 vs. AN2	1;103	1.065	0.356	0.010	0.910	0.664	0.009

Comparisons of A) interindividual variation, B) composition between studied groups based on Betadisper tests and PERMANOVA, respectively. Tests were conducted using relative abundance-based (Bray-Curtis) and prevalence-based (Jaccard) dissimilarities. Values of (pseudo-) F statistics (F), associated degrees of freedom (df), resulting probability values (p), and proportions of explained variance (R^2^) are shown. Significant values are in bold.

**Figure 4.**
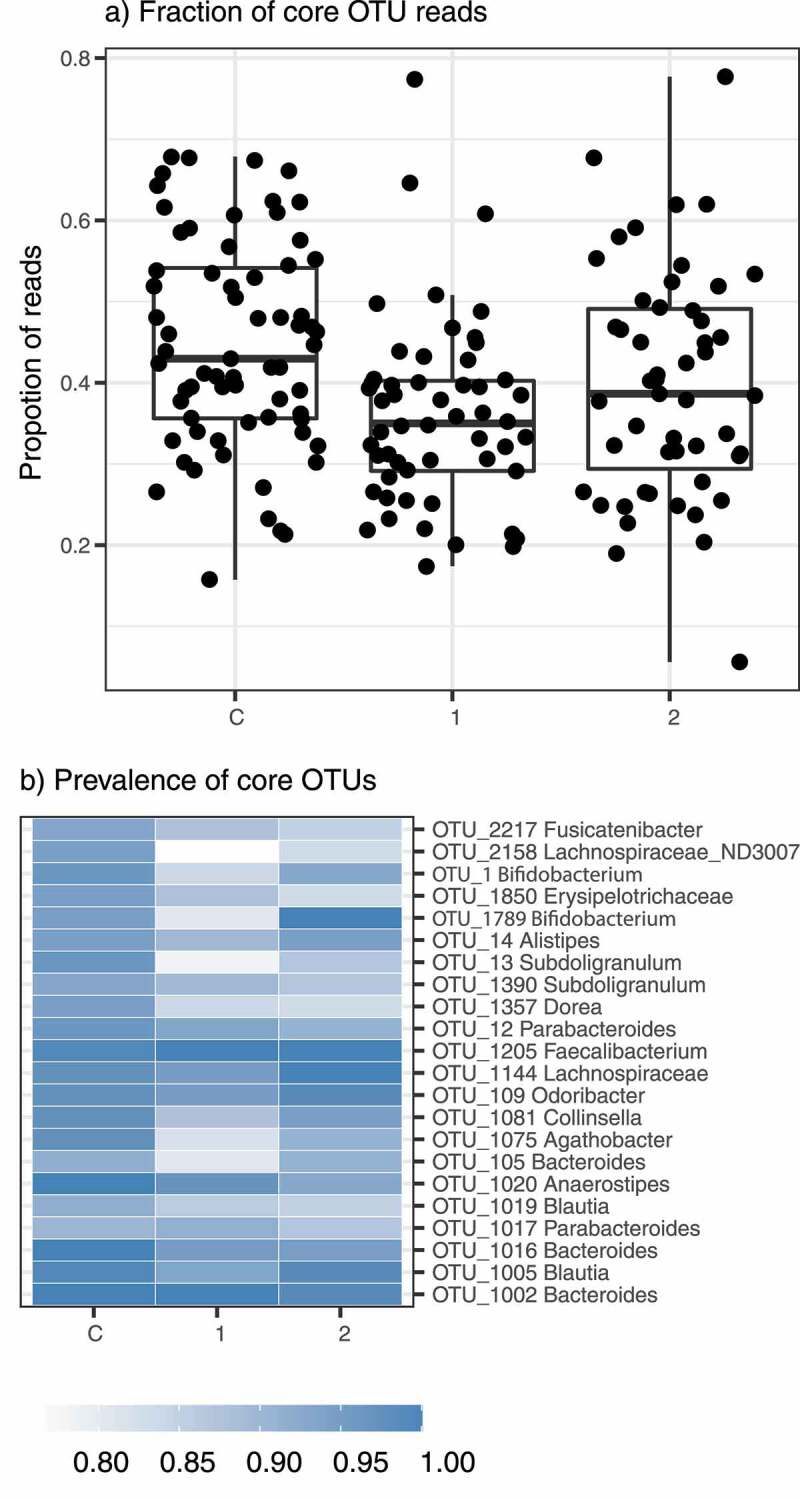
Bacterial core microbiota in the studied groups. A) Proportion of reads corresponding to core bacterial OTUs (i.e. detected in >90% samples) in each studied group and B) heatmap showing their prevalences

It is generally assumed that indispensable microbiome functions are facilitated by so-called ‘core microbiota’ (a set of highly prevalent bacteria^21^), and that depletion of these keystone species may disturb ecosystem services provided by microbiota to the host. Here we observed core microbiota depletion signs in AN1 and AN2, which was putatively driven by increased interindividual variation in AN1 and AN2. Specifically, there were 21 core bacterial OTUs shared among >90% of control individuals, but only 14 OTUs in the case of AN2 and 9 OTUs in AN1. Moreover, these core bacteria (22 unique OTUs in total) represented on average 45% of all reads in control microbiota profiles, but only 40% and 36% in the case of AN2 and AN1, respectively (ANOVA: F_(2,169)_ = 7.041, *p* = 0.00116, Figure 4).

**Figure 5.**
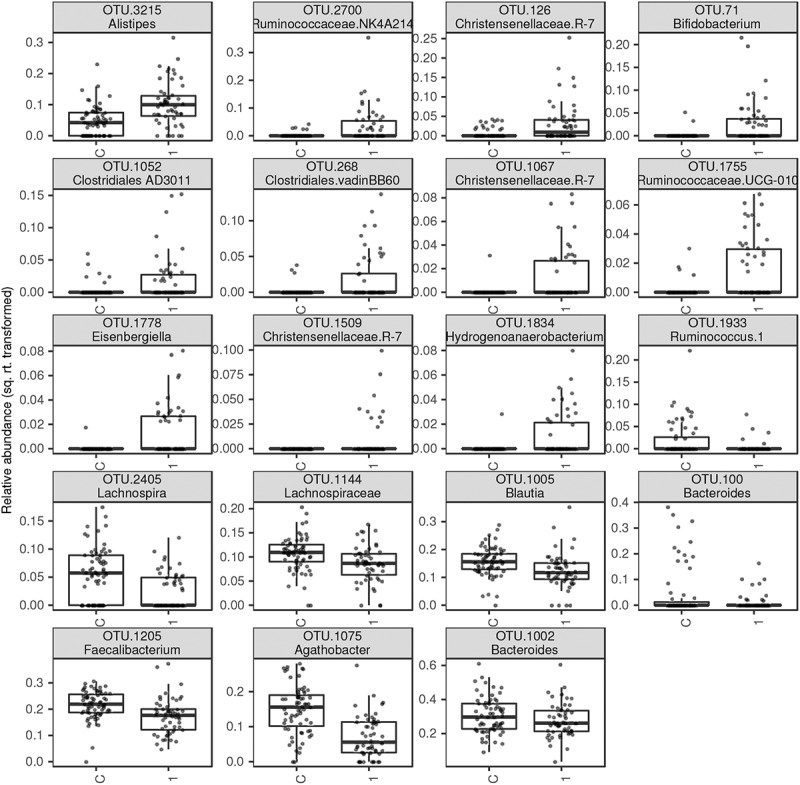
Relative abundances of bacterial OTUs (squared-root transformed) that varied, according to DESeq2 analyses (FDR < 0.05), between control samples and AN1

DESeq2 analyses identified 11 OTUs that were overrepresented (taxonomically belonged to *Alistipes, Clostridiales, Christensenellaceae*, and *Ruminococcaceae*) and eight that were underrepresented (*Faecalibacterium, Agathobacter, Bacteroides, Blautia*, and *Lachnospira*) in AN1 compared to control samples (Figure 5). Simultaneously, only a single OTU (*Megapshaera*) exhibited a significant abundance increase, and no OTU exhibited a significant abundance decrease in AN1 compared to AN2. Interestingly, *Methanobrevibacter smithii* abundance that was based on previous reports increased in anorectic patients,^8,10,11,13^ exhibited approximately twice greater abundance in AN1 (average relative abundance = 0.0042) and AN2 (0.0036) compared to the control group (0.0017). However, this difference was not supported by DESeq2 analyses (*p* = 0.3433, adjusted *p* = 0.6616). Taxonomic features detected by DESeq2 pertaining to bacterial genera abundances (or greater taxonomic ranks) exhibited great consistency with OTU-level results (Fig. S2). Nevertheless, there were a subset of OTUs not recovered by genus-level analyses (e.g. *Anaeroplasma, Dorea, Anaerotruncus*, and *Hydrogenobacterium*) and vice versa (e.g. *Alistipes, Ruminococcaceae NK4A214, Christensenellaceae R−7*, and *Ruminococcus 1*). This discrepancy may be partly explained because some OTUs within individual genera varied in their response to anorexia. For example, only a single *Alistipes* OTU (of 14 detected) (related to *A. finegoldi/A. onderdonkii* according to phylogenetic placement analyses (Fig. S3)) exhibited a significant abundance increase in AN1, whereas the others exhibited no significant variation or even the opposite pattern.

### Bacterial community composition association with EDE-Q scores, BMI, hyperactivity, and anorexia duration

We did not find any association between EDE-Q scores, BMI, hyperactivity, or disease length, and any of the tested alpha diversity measures (*p* > 0.1 for all comparisons). Furthermore, alpha diversity changes during hospitalization (i.e. the difference in alpha diversity between AN1 vs. AN2) did not correlate with the BMI and EDE-Q score changes, reported outcome, or hospitalization length (*p* > 0.2 for all comparisons).

Using data from AN1, we did not find any effect of EDE-Q scores, BMI, hyperactivity, or disease duration on the gut bacteriome composition (db-RDA: *p* > 0.10 for Bray-Curtis and Jaccard divergence). Furthermore, the magnitude of gut bacteriome changes during hospitalization was not associated with changes in EDE-Q scores or BMI changes (*p* < 0.2 for both Jaccard and Bray-Curtis dissimilarities). However, the divergence in gut bacteriome between AN1 vs. AN2 was positively correlated with log-scaled hospitalization length (Bray-Curtis: F_(1,46)_ = 6.980, *p* = 0.011, Jaccard: F_(1,46)_ = 11.241, *p* = 0.002). Simultaneously, positive outcome (i.e. outcome scores = 1 or 2) was reported in patients exhibiting less pronounced bacterial changes after statistical control for the hospitalization length (Bray-Curtis: F_(2,46)_ = 4.579, *p* = 0.015, Jaccard: F_(2,46)_ = 6.878, *p* = 0.002; Fig. S5). Finally, the hyperactive patients exhibited smaller changes in OTU relative abundances compared to patients with no signs of hyperactivity (Bray-Curtis: F_(1,46)_ = 5.2473, *p* = 0.02672). Conversely, hyperactivity did not predict magnitude of changes in OTU prevalences (Jaccard: F_(1,46)_ = 0.4914, *p* = 0.4869; Fig. S5). However, subsequent mixed models did not detect any OTU exhibiting inconsistency in abundance changes between hyperactive vs. non-hyperactive patients (i.e. hyperactivity × group identity [AN1 vs. AN2] interaction; FDR > 0.05 in all cases; Fig. S5). This suggests that greater changes in non-hyperactive patients were of stochastic nature with rather unpredictable consequences for the bacteriome content.

### Metagenomic prediction

**Figure 6.**
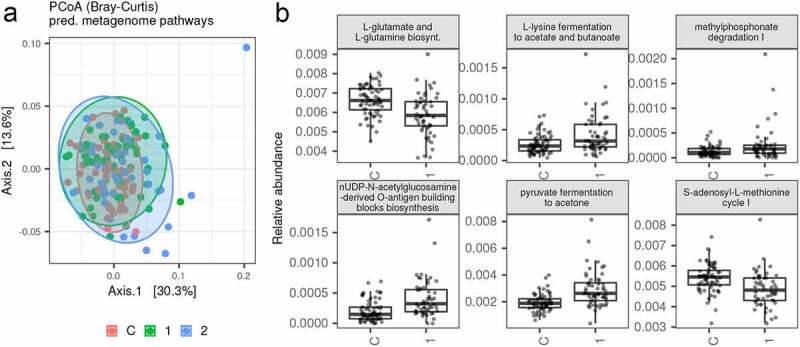
A) PCoA showing variation in relative abundances of predicted metabolic pathways between controls vs. AN1 vs. AN2, and B) Relative abundances of bacterial metagenomic pathways (squared-root transformed) that varied, according to DESeq2 analyses (FDR < 0.05), between control samples vs. AN1

We used PICRUSt2 pipeline to infer variation in metagenome functions based on 16S rRNA profiles, and multivariate analyses on predicted relative abundances of functional pathways recapitulated patterns observed at the OTU level. Specifically, AN1 and AN2 exhibited increased interindividual variation in the predicted metagenome content compared to controls (betadisper: *p* = 0.001 in both cases), and pair-wise PERMANOVA analyses suggested that the predicted metagenome content differed between controls vs. AN1 and AN2 (*p* = 0.017 and 0.003, respectively; Figure 6). Consistent with OTU-level analyses, hospitalization did not result in systematic gut bacteriome function changes (PERMANOVA: *p* = 0.395), nor in changes in their interindividual variation (betadisper: *p* = 0.224). Using DESeq2, we identified two overrepresented and four underrepresented predicted metabolic pathways in controls vs. AN1 (Figure 6).

### Fungal communities

The ITS profiles dataset included a high percentage of non-fungal reads (50%). After the elimination of these non-target taxa, we obtained 488 fungal OTUs represented by 2,346,476 high-quality sequences with an average sequencing depth per sample of 13,642 reads (range = 78–63,662). We excluded 16 samples that comprised a fewer number of fungal reads (<1000) from all downstream analyses.

Fecal samples in our study included 13.60 (range = 1–99) different fungal OTUs on average, as predicted by Chao 1 estimates, but there was no significant difference in alpha diversity between the studied groups (LMM: *p* > 0.7 for all alpha diversity measures). The fungal communities composition did not correlate with the bacterial profile composition, neither for all sample analyses in our dataset (Mantel test, r = −0.038, *p* =0.77 for Bray-Curtis and r = −0.0395, *p* = 0.856 for Jaccard dissimilarities) nor for separate sample analyses for each category (Mantel test: *p* > 0.5 for all combinations of categories vs. dissimilarity measures). Consequently, our data does not provide clear evidence that variation in gut bacteriome follows changes in gut mycobiome and/or vice versa.

**Figure 7.**
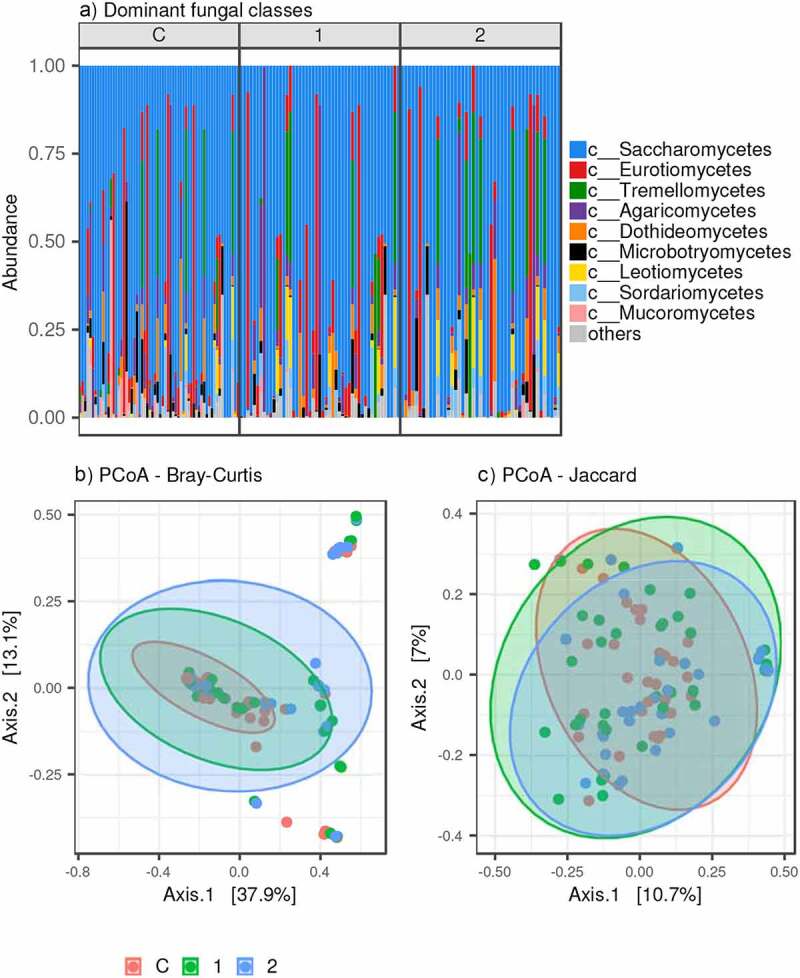
Fungal microbiota variation in the three studied groups. A) Dominating fungal class proportions (represented by >1% of reads); PCoA for B) Bray-Curtis and C) Jaccard dissimilarities between samples

Fungi from class Saccharomycetes dominated in fungal profiles of most samples (68% of reads on average, dominated by genus *Saccharomyces, Candida*, and *Nakaseomyces*). However, in the samples subset, we observed representatives of class Eurotiomycetes (10% of reads, represented by *Penicillium* and *Aspergillus*), mushroom-forming fungi Agaricomycetes (5% of reads, represented by *Agaricus* and *Boletus*), and Tremellomycetes (*Solicoccomyza, Cryptococcus*, and *Naganishia*, Figure 7, S1).

Unlike the bacterial dataset, we did not find any systematic shifts in fungal profiles composition between the studied groups (PERMANOVA: *p* >0.2 for both Bray-Curtis and Jaccard dissimilarities; Fig. S6), and interindividual variation in the fungal profile composition was the same within each group (Betadisper: *p* >0.2 for both Bray-Curtis and Jaccard dissimilarities).

Only three fungal OTUs of lesser prevalence exhibited abundance differences between controls vs. AN1. OTUs belonging to *Nakaseomyces* were overrepresented in AN1, whereas *Mucor* and *Naganishia* OTUs were more abundant in control samples, according to DESeq2 analyses (Fig. S6). These differences, however, mirror the fungal composition in only a few individuals. Furthermore, there were no changes in fungal OTUs and genera between AN1 vs. AN2.

### Neurotransmitter and SCFA levels

Fecal concentrations of assorted neurotransmitters were measured by mass spectrometry (MS). In AN1 samples decreased GABA and dopamine levels were found. Similarly, decreased serotonin level was detected in the AN1 and AN2 groups, but was significant only in renourished patients (Table 7). Tyramine, kynurenine, and hydroxytryptophan concentrations did not vary between groups (control, AN1, AN2), and they did not change during the course of hospitalization.

**Table 7.** Neurotransmitter and SCFA levels in healthy controls and patients

				p-value	%	p-value	%	p-value	%
Variable	Control	AN1	AN2	AN1 vs. AN2		AN1 vs. C		AN2 vs. C	
GABA	1.8 (1.47, 3.29)	1.39 (0.997, 2.41)	1.53 (1.02, 3.31)	0.372	10.1	0.014*	−22.8*	0.190	−15.0
Tyramine	3.47 (2.07, 6.45)	3.45 (1.54, 6.66)	2.36 (1.24, 5.92)	0.480	−31.6	0.796	−0.6	0.385	−32.0
Serotonin	0.482 (0.238, 0.846)	0.332 (0.234, 0.602)	0.266 (0.214, 0.443)	0.058	−19.9	0.371	−31.1	0.012*	−44.8*
Dopamine	0.102 (0.046, 0.267)	0.044 (0.017, 0.096)	0.044 (0.017, 0.256)	0.596	0.5	0.025*	−57.1*	0.084	−56.9
Kynurenine	0.064 (0.047, 0.103)	0.063 (0.042, 0.087)	0.063 (0.051, 0.095)	0.944	−0.9	0.997	−0.9	0.986	−1.9
5-HTP	0.047 (0.027, 0.07)	0.043 (0.032, 0.064)	0.041 (0.028, 0.065)	0.539	−4.4	0.856	−7.7	0.922	−1.,8
Butyrate	2.01 (1.37, 2.8)	1.2 (0.738, 2.13)	1.38 (0.944, 2.29)	0.263	15.0	0.038*	−40.3*	0.298	−31.3
Propionate	2.08 (1.55, 2.94)	1.84 (1.06, 2.34)	1.59 (1.11, 2.4)	0.897	−13.6	0.108	−11.5	0.028*	−23.6*
Acetate	19 (13.7, 25.9)	14 (7.2, 18.8)	12 (8.55, 17.4)	0.286	−14.3	˂0.001***	−26.3***	0.013*	−36.8*

The comparison of a control group with both AN1 or AN2 was analyzed by one-way ANOVA with Dunnett’s test. The changes during hospitalization of patients with AN were tested by paired t-test. *p˂0.05, ***p˂0.001. nControl = 67, nAN1 = 49–53, nAN2 = 39–51. Data are also presented as a percentage change relative intensity between groups. Significant changes are in bold. GABA – gamma-aminobutyric acid; 5-HTP – 5-hydroxytryptophan

Fecal SCFA levels were assessed by nuclear magnetic resonance (NMR). Patients (AN1) exhibited a decreased butyrate level, which slightly increased after renourishment. Propionate abundance was slightly reduced in patients after hospitalization (AN2). Acetate concentration was much less in patients with AN at both sampling intervals. We did not detect any significant changes in neurotransmitters and SCFA levels after renourishment (Table 7).

### Bacterial community composition association with SCFAs, neurotransmitters concentration, biochemical and anthropometric parameters

**Figure 8.**
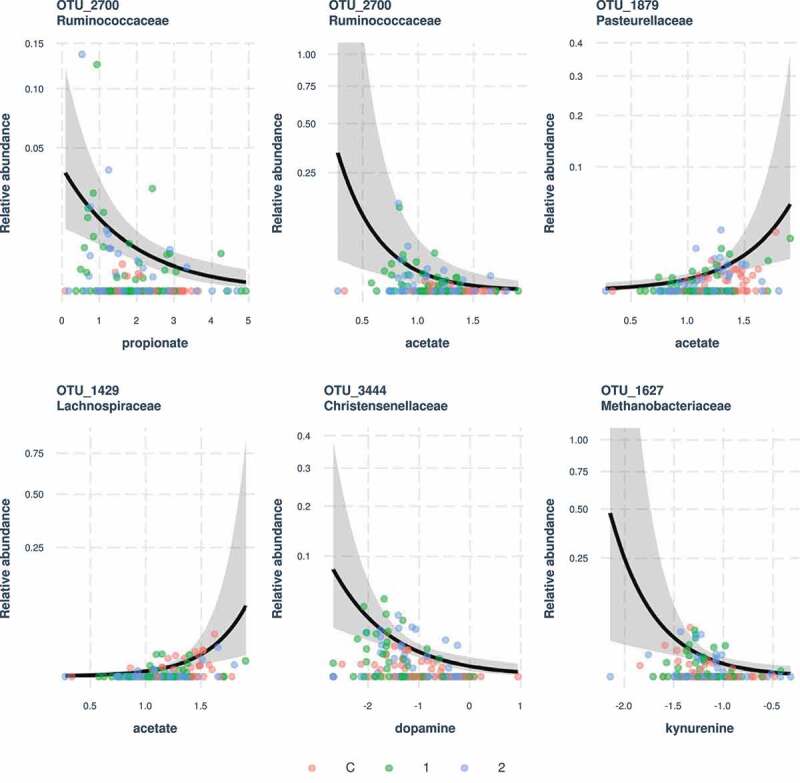
Significant associations between bacterial OTU abundances and concentrations of SCFAs or neurotransmitters. Predictions and 95% confidence intervals for negative binomial generalized linear mixed models (GLMMs) are shown

The relative abundance of OTU_2700 *Ruminococcaceae NK4A214* was negatively associated with propionate and acetate concentrations, while acetate levels were positively associated with the abundance of two OTUs (OTU_1879 *Pasterullaceae* and OTU_1429 *Lachnospiraceae*). Similar analyses that focused on links between bacterial OTUs and neurotransmitter concentrations found a negative effect of dopamine on OTU_34444 *Christensenellaceae* and kynurenine on OTU_1627 *Methanobacteriaceae* (Figure 8). Genus-level analyses did not find any association with SCFA concentrations, but abundances of four genera were negatively linked with dopamine concentrations and three with kynurenine concentrations (Fig. S4). These effects could not arise as an inter-group abundances variation by-product of these taxa, as our models took this potentially confounding factor into account.

Finally, we found only two significant associations between bacterial OTU/genera abundances and any of the biochemical and anthropometric parameters. Namely, relative abundance of *Ruminiclostridium*_9 was negatively related to α1 globulin (g/l) (GLMM slope [± S.E] = −3.48562 ± 0.60499, FDR < 0.0001), while there was positive association between *Dialister* and γ globulin (g/l) (GLMM slope [± S.E] = 1.3001 ± 0.1815, FDR < 0.0001).

## Discussion

To the best of our knowledge, our study for the first time reports the combined intestinal microbiota composition, SCFAs, and neurotransmitter levels together with biochemical, anthropometric, and psychometric profiles in a substantial number of patients with AN before and after hospitalization in comparison to normal-weight healthy participants.

Patients with severe or extreme AN (an average BMI of 14.4 kg/m^2^ at admission), increased their BMI by 2.18 kg/m^2^ during the realimentation (Table 1). We confirmed the literature findings that BMI increase can be predicted by many bio-psycho-social factors (Table 4). However, only the history of adulthood stress (surprisingly not the childhood stress known to influence the early childhood microbiome) negatively influenced the BMI gain in patients with AN (Table 3). Stress activates the hypothalamic-pituitary-adrenal (HPA) system as well as the sympathetic nervous system (SNS), and thus affects the immune system. A chronically upregulated HPA axis and SNS are often associated with AN co-morbidities, like obsessive-compulsive disease, depression, anxiety, post-traumatic stress disorder, and others.^22^ Since weight and nutritional restoration are key conditions for AN recovery, our criteria for positive outcome are very complex, expressed by global impression at discharge, including BMI gain (and motivation to maintain it), the patient’s attitude, body image perception, and awareness of relapse risk factors. In our final study cohort, we did not detect the negative outcome. However, seven patients with AN discharged earlier due to their treatment violation or their drop-out were not included into the final analysis. These patients would most likely report the unchanged or negative outcome. The prediction model confirmed that positive patient outcome can be predicted to a certain level by several clinical parameters (Table 5), similarly to Wales et al., who demonstrated that greater entry BMI and early weight gain predicted a positive treatment outcome in patients with AN.^23^

We hypothesized that microbial diversity and composition in patients with AN differ from healthy individuals, and better understanding will improve AN course, prediction, and therapy implementation. Of the measured alpha-diversity parameters only the Chao 1 index was increased in patients before renourishment, reflecting their interindividual variation. The OTUs number exhibited a comparable yet nonsignificant pattern, and the qualitative Shannon diversity did not vary among the studied groups (Figure 1). A similarly large microbiome study of patients with AN (n = 55) found no differences in the number of observed species and Chao 1 index.^13^ Three other smaller studies describe decreased alpha diversity and microbial richness in patients with AN.^7,12,15^ Since the results vary between studies, it is difficult to unequivocally interpret the modification of alpha diversity parameters during AN. Weight gain in our patients with AN led to Chao 1 index modification and reached healthy control values. Mack et al. described increased species richness in patients with AN after weight gain, although there was no difference when compared to healthy controls.^13^

The control group exhibited less interindividual variation, which was manifested by core microbiota depletion, as well as a systematic difference in gut bacteriome compared to patients with AN (Table 6). Greater interindividual variation of patients with AN can be explained by the so-called “Anna Karenina principle,” explaining that dysbiotic individuals vary more in microbial community composition than healthy individuals due to stochastic microbiota response to a disequilibrium state induced by stressors. Such effects on the microbiome are common, important, and they are often associated with host health impairment.^24^

Both AN1 and AN2 exhibited increased interindividual variation of gut bacteriome composition compared to controls, which was putatively linked with partial depletion of core bacteriome OTUs (Figure 4). Most AN studies describe microbial alteration at the genera, class, or even phylum level. However, differences in specific OTUs rather than in the specific microbiome communities may address their important role in illness pathology.

Patient renourishment led to minor bacterial composition changes (Table 6), which are supported by the positive correlation of hospitalization length and bacteriome divergence of AN1 vs. AN2. The bacteriome of patients after weight gain was still more similar to the bacteriome of patients at admission than to the bacterial composition of healthy controls, which is in accordance with Mack et al.^13^ We detected a significant change in a subset of OTUs that did not correspond to the results from a separate genus-level analysis; however, these OTUs may play an important role in AN pathophysiology. For example, only a single *Alistipes* OTU (out of 14 detected) exhibited a significant abundance increase in AN1. This OTU_3215 is related to *A. finegoldi* and *A. onderdonkii* (Fig. S3). Different strains of the *Alistipes* genus were shown to have unique physiological roles associated with different diseases and disorders.^25^ Since *Alistipes* can hydrolyze tryptophan (serotonin precursor) to indole and thus decreases serotonin availability, *Alistipes* increased abundance can disrupt the gut-brain axis. Further, a decrease in serotonin is associated with depression.^26^

Only one *Faecalibacterium* OTU exhibited a significant abundance decrease in AN1. Similarly, altered microbiota with overexpressed *Alistipes* and decreased *Faecalibacterium* levels were found in patients with depression,^26^ a common AN comorbidity. *Faecalibacterium* levels are reduced in many human diseases and disorders, including patients with AN.^7^ Studies examining gut *Faecalibacterium* suggest that its greater abundance is associated with a healthier state and proposes its potential therapeutic usage. Further observed overrepresented OTUs in patients with AN taxonomically belonged to *Christensenellaceae*. The *Christensenellaceae* family was enriched in subjects with a lesser BMI.^27^ Moreover, the *Christensenellaceae* family is heritable and host genetics influence the human gut microbiome composition.^27^ Conversely, one of the less represented species in AN1 was *Agathobacter*. There is a discrepancy in the taxonomic annotation of *Agathobacter, Roseburia*, and *Eubacterium*. Because the different reference databases can annotate the same sequence with different species, we presume that decreased *Agathobacter* levels in patients with AN in our study correspond to the decreased *Roseburia* levels in other studies.^11,13,14^

These findings indicate that different OTUs corresponding to the same bacterial genus can be associated with different features. We believe that abundance changes at the OTUs level reflect more their importance than changes at the genus level.

Only a few positive or negative correlations between bacterial OTUs or genus abundances and SCFAs or neurotransmitters amount were found (Figure 6, Fig. S4). This can be explained by the overlapping production of these molecules by many bacterial species. Moreover, since the majority of SCFAs produced in the colon are absorbed by the gut mucosa, stool SCFAs quantification does not accurately reflect the bacterial production level.^28^

Except for the positive correlation of bacteriome changes with hospitalization length, we did not find any association of bacterial community composition and diversity with disease duration, BMI, and EDE-Q scores. However, besides the greater interindividual variation in gut bacteriome compared to controls, patients with AN also exhibited greater interindividual variation in the metagenome content, suggesting altered bacteriome functions (Figure 7).

Variations in predicted metabolic pathways associated with AN were not described in any other microbiome study. In AN1 samples, we identified an underrepresented S-adenosyl-L-methionine cycle I pathway (Figure 7). In cells, about 80% of the L-methionine pool is converted to S-adenosyl-L-methionine (SAM), which is the major methyl donor. After the methyl group donation, SAM is converted to S-adenosyl-L-homocysteine, which can be recycled back to SAM via this cycle. Deficiency in SAM production and subsequent DNA methylation, an important epigenetic mechanism, is connected with severe neuro-psychiatric diseases.^29^ Besides, SAM is required for some neurotransmitter synthesis, such as serotonin, dopamine, and norepinephrine.^29^ Interestingly, the use of SAM as a dietary treatment leading to modulation of patient methionine metabolism via altered nutrient availability showed promising results in depression disorder treatment.^30^

None of the existing microbiome studies on patients with AN analyzed fungal communities together with bacterial composition. There is growing evidence emerging that host fungal communities could also be linked with various diseases. However, during the mycobiome analysis, we found neither significant differences in mycobiome alpha diversity between patients with AN and controls, nor any correlation of the fungal composition with bacterial profiles. However, fungi constitute less than 0.1% of the human gut microbiome.^5^ Their composition and abundance are strongly affected by food, and the composition is not stable over time.^5^ We detected two different phylotypes across all studied groups. The first phylotype was dominated by Saccharomycetes, and the second was comprised of various fungal species. The absence of abundant Saccharomycetes in the second group most likely led to the PCR amplification of rare species, and subsequently to their greater diversity. During the gut mycobiome assessment in the frame of the Human Microbiome Project, the most abundant human gut genera include the yeasts *Saccharomyces, Malassezia*, and *Candida*.^5^ Interestingly, this study recognized *Saccharomyces* and *Candida* as prevalent members of the gut mycobiome, but did not identify *Malassezia*. Similarly, this discrepancy was also found by Hoffmann *et al*. related to mycobiome changes associated with diet.^31^ One explanation could be the use of primers amplifying the ITS1 region, when primer sequence mismatches may not allow optimal amplification of *Malassezia* DNA. However, we used primers amplifying the ITS2 region as in the Human Microbiome Project, although the primer sequences differed. Potentially, *Malassezia* was not identified in our dataset due to different diet or geographic location. The only overrepresented fungal species in AN1 was *Nakaseomyces*, and its clade includes various human pathogens.^32^ It seems that balanced fungal representation preventing overcolonization during severe dysbiosis is most likely more important than specific taxonomic abundance. Previously, great abundance and variability of fungal species were found in one patient suffering from severe and enduring AN.^33^

The gut-brain axis is increasingly connected with central nervous system health. Since gut microbiota may produce various neurotransmitters as well as neuro-reactive microbial metabolites, including SCFAs, we aimed to assess the levels of assorted neurotransmitters and individual SCFAs in AN patient stools. We found significantly decreased levels of GABA and dopamine, and slightly decreased serotonin levels in stools of patients with AN compared to healthy controls (Table 7). Individuals suffering from AN have altered brain serotonin and dopamine pathways.^34^ There are indicators that altered serotonin function contributes to anxiety in patients with AN.^34^ Similarly, changed GABA levels were implicated in a variety of mental illnesses, including anxiety and depression.^35^ Interestingly, the levels of the serotonin precursor 5-hydroxytryptophan (5-HTP) as well as kynurenine levels, an alternative tryptophan metabolite, were comparable in all study groups, suggesting that probably only part of 5-HTP is converted to serotonin in patients with AN. Neurotransmitter levels are generally assessed in the brain. Reduced neurotransmitter levels in individuals with AN tend to normalize with recovery.^34,35^ We analyzed the levels of various neurotransmitters in the stools of patients with AN and healthy controls, and these did not change after the realimentation (Table 7). This raises the question whether altered metabolite levels in the gut can truly effectively influence gut-brain communication and eventually modify the impact of medications. SCFAs are extensively studied as molecules associated with gut microbiota effect host energy metabolism and appetite.^36^ We found reduced butyrate and acetate levels in patients with AN, which were not altered after weight gain (Table 7). SCFA abundance is inconsistently reported in AN studies. While one study showed reduced concentrations of acetate and propionate,^9^ another found decreased butyrate and propionate concentrations.^11^ A longitudinal study by Mack *et al*. observed a slightly decreased butyrate level in patients with AN, which did not change after weight gain, similar to our results.^13,33^

Only a few positive or negative correlations between bacterial OTUs or genus abundances and SCFAs or neurotransmitters amount were found (Figure 8, Fig. S4). This can be explained by the overlapping production of these molecules by many bacterial species. Moreover, since the majority of SCFAs produced in the colon are absorbed by the gut mucosa, stool SCFAs quantification does not accurately reflect the bacterial production level.^28^ The correlation analysis of fecal metabolites and bacterial composition in patients with AN revealed metabolite consumption by the intestinal microbiota outweighs their production.^15^

This longitudinal study provides biochemical, anthropometric, psychometric, comprehensive microbiome, and targeted metabolomic data from a large cohort of patients with AN compared to healthy controls. The results are based on quality sequencing microbiome data and underline the importance of individual OTUs rather than taxonomical genus abundance. Since approximately 10% of patients with AN are males, and AN has an onset in adolescence, our cohort represented only by adult women can pose a potential weakness.

Further, the use of antidepressants and other medications can alter the microbiome as well as neuroactive microbial metabolites and neurotransmitters production, therefore, we cannot rule out that medication use might influence our results. Another clear limitation of the metagenomics study is host microbiome variability, which can change in response to diet or other environmental factors. Alternatively, the host microbiome is susceptible to its preventive or therapeutic target modification.

Although therapeutic renourishment led to increased body mass index (BMI) and improved psychometric parameters, SCFA and neurotransmitter profiles as well as microbial community compositions did not change substantially. This can be potentially explained by the only partial weight gain of patients with AN, which mostly did not reach healthy control values during hospitalization. To achieve full recovery (normalization of the fat mass, BMI 19–25, menstruation, etc.), patients are offered post-hospitalization programs.

## Patients and methods

### Participants

The study was performed in accordance with the Declaration of Helsinki, and it was approved by the Ethics Committee of General University Hospital in Prague. All participants signed an informed consent form.

Fifty-nine patients with restrictive AN (age 23 (19, 27) years), BMI 14.4 (13.4, 15.9) kg/m^2^, shown as median with quartiles, were recruited from the in-patients of the Center for Eating Disorders at the Psychiatric Clinic of the First Faculty of Medicine of Charles University and the General University Hospital in Prague (Table 1). Patient severity of AN was diagnosed according to the Diagnostic and Statistical Manual of Mental Disorders, 5^th^ Edition, American Psychiatric Association, 2013 (none, mild, moderate, severe, and extreme). Only clinically stable patients without psychosis or current abuse on psychoactive substances were included. Seven patients with AN terminated the therapy prematurely. Hospitalization duration was 51 (28.5, 62.5) days (Table 1). At the patients’ discharge, outcome scoring, representing the global clinical impression, was determined (no change, slight improvement, significant improvement, slight worsening, and significant worsening). Patients who took some medication before hospitalization took the same medication during AN treatment. 32 patients of 59 total participants (50.9%) were using antidepressants (majority SSRI type), 16 patients (25.4%) antipsychotics (mainly evidence-based Olanzapine), and 32 patients (50.9%) were taking other medications (anxiolytics or hypnotics, hormonal substitution, vitamins or analgetics, Omeprazole, digestive aids).

Sixty-seven healthy female controls were recruited for the study, comprising university students, office workers, and university employers. Healthy controls were screened for a hidden eating disorder by the SCOFF Questionnaire. The controls were healthy weight Czech women (age 24 (22, 28.5) years) with BMI 21.9 (19.9, 23.7) kg/m^2^ (Table 1).

Exclusion criteria for all participants were pregnancy, diabetes, any severe active infection, and various chronic diseases. Additionally, controls did not suffer from any subtypes of AN or BN, or other psychiatric disorders.

Anthropometric measurements (i.e., height, weight, fat percentage, hipline, and waistline) were supervised and taken by nursing staff and occurred on bioimpedance scales (TANITA, Japan). Blood samples were collected from all participants from the cubital vein early morning; from patients with AN there were two collections – at admission and discharge. On the same day, stool samples of all participants were collected and immediately frozen at −80°C. Participants were asked not to drink alcohol, coffee, black tea; not to eat chocolate, products containing cacao, bananas, nuts; and not to take probiotics or aspirin two days before the stool collection.

### Biochemical analysis of blood samples

Blood samples of controls and AN patients at both intervals (before and after hospitalization) were used for determining serum levels of albumin, total protein, alfa-, beta-, and gamma-globulins; immunoglobulins IgA, IgG, IgM, and IgE; CRP, cholinesterase, triacylglycerols, TSH, and fT4 (Agilab, Czech republic; Table 1). Further, serum was used for IL-6, IL-17, and TNF-α levels assessment by ELISA (Human IL-6, IL-17, and TNF-α Quantikine HS ELISA kit, Bio-Techne R&D Systems, USA; Table 1).

### Questionnaires

All participants completed questionnaires (AN patients at admission and discharge) addressing hyperactivity (none, present, significant), disease duration (months), menarche (age), sleeping habits (time of getting up, going to sleep, sleep length), exercise activity (hours per week), number of daily meals, allergy, history of a stressful event (none, present until 3 years of age, present in adolescence, present in adulthood), antidepressant and other medication, another somatic or psychiatric diagnosis, psychiatric disorders heredity, childbirth type, menstruation presence, employment/education (disability pension, university education, secondary school education, basic school education, secondary school student, university student).

### Eating disorder examination (EDE-Q)

AN patients completed the EDE-Q 6.0 within 24 hours of admission.^37^ The EDE-Q 6.0 is a 28–item measure derived from the Eating Disorder Examination (EDE).^38^ The 22 items together comprise four subscales, assessing restraint, shape concerns, weight concerns, and eating concerns over the previous 28 days. They were scored using a 7-point rating scale (0–6). The sum of each subscale was averaged to provide subscale scores. A global score was calculated by summing and averaging the subscale scores. Greater scores indicate greater ED psychopathology. Another six questions assessed the frequency (number of times or days) of specific eating behavior, such as objective binge eating, self-induced vomiting, laxative use, or excessive exercise, over the last 28 days. These are not included in the subscale scores. The internal consistency reliability of the EDE-Q was measured with Cronbach’s α coefficients. Data are presented as mean scores on the EDE-Q global and sub-scale scores.

### Statistical analysis

For the basic measured parameters, comparison of the three studied groups (controls, AN1 – patients at admission, and AN2 – patients at discharge) was analyzed by the Kruskal–Wallis Z test followed by Dunn’s multiple comparisons with Bonferroni correction. The evaluation of changes during hospitalization (calculated as the values at hospitalization end and values at hospitalization beginning) were evaluated by Wilcoxon’s paired test corrected for ties. Besides the Bonferroni correction for between-group differences we also completed the Bonferroni correction for multiplicity for Kruskal–Wallis test and Wilcoxon’s test respecting the 27 biochemical and anthropometric parameters under investigation.

To obtain data symmetry and homoscedasticity of non-Gaussian distributed metabolomic data, the original continuous variables were transformed by power transformation. The comparison of a control group with both the AN1 or AN2 groups was analyzed by one-way ANOVA with the Dunnett’s test. The changes during patients’ renourishment were tested by paired t-test.

The relationship between variables and individual predictors was evaluated by multivariate regression (MR) with a reduction of dimensionality known as orthogonal projections to latent structure (OPLS). OPLS for one predicted variable allows great intercorrelation determination and enhances the model predictivity. MR without dimensionality reduction was employed for a specific correlation with one predictor (uncorrelated with other variables). OPLS is capable of coping with the problem of severe multicollinearity (great intercorrelations) in the predictors matrix, while multiple regression fails to evaluate such data. Assessing patient outcome after renourishment utilized the logarithm of the ratio of the probability that the patient’s psychopathology improved to the probability that not. The original probabilities of the negative outcome were transformed to logarithms of the likelihood ratio (logarithm of the ratio probability of negative outcome/(1-probability of negative outcome)) and this (transformed) parameter was used as a dependent variable in both the OPLS model, as well as in MR analysis.

The correlation between individual EDE-Q subscales was analyzed by Pearson’s correlations after the power transformation of data. The internal consistency reliability of the EDE-Q was measured with Cronbach’s α coefficients.

Statistical software Statgraphics Centurion 18 Version 18.1.06 from Statgraphics Technologies, Inc. (The plains, VA, USA) was used for Box-Cox transformations, ANOVA testing, while the OPLS and MR analyses were performed using the software SIMCA P+ Version 12.0.0.0.

### Gut microbiota analysis

Genomic DNA was isolated from stool by DNeasy PowerSoil Kit (Qiagen). The total extracted genomic DNA (gDNA) from stool samples was used for high throughput sequencing (HTS, Miseq platform, Illumina) of the bacterial V3-V4 region of the 16S rRNA gene, and fungal ITS2 region. Besides the isolated gDNA, ZymoBIOMICS^TM^ microbial community standard and Standard II (log distribution), as well as ZymoBIOMICSTM microbial community DNA standard and Standard II (log distribution), were used to assess the performance of entire metagenomic workflows (Zymo research). The original standards’ microbial composition and the obtained sequencing data are shown in Table S3,4. Two sets of specific primers with barcodes (342 F/806 R primer for 16S rRNA^39^ and ITS7/ITS4 for fungal ITS^40^) were used in PCR using HiFi HotStart Ready Mix (Roche). PCR reactions were carried out with 25 (bacteria) and 30 (fungi) cycles. Triplicates of the amplicons were pooled, normalized with the SequalPrep™ Normalization Plate Kit (ThermoFisher Scientific), and pooled and concentrated on a Concentrator 5301 (Eppendorf) for approximately 3 h at 30°C under vacuum. The resulting volume of each library was purified using the DNA Clean & Concentrator kit (Zymo Research) and ligated with sequencing adapters (TruSeq DNA PCR-free LT Sample Preparation Kit, Illumina) using KAPA HyperPlus Kit, (Roche). Concentrations of libraries with ligated adapters were validated by a KAPA Library Quantification Kit (Illumina). The final libraries were pooled in equimolar concentrations, and sequenced. The amplicons were sequenced on an Illumina MiSeq using a Miseq Reagent Kit v2 (Illumina).

### Bioinformatic pipeline

Fastq files produced by Illumina Miseq were demultiplexed and primers were trimmed by *skewer* software.^41^ Using *dada2*^42^ we eliminated low-quality sequences (expected number of errors per read >1), denoised quality-filtered fastq files, and constructed an abundance matrix (OTU table) representing reads counts for individual haplotypes in each sample. Next, we identified chimeric haplotypes using *uchime*^43^ and the gold.fna database (in the case of bacterial data) or UNITE database^44^ (in the case of fungal data) and eliminated them from the OTU table. Using Procrustean analyses, we checked for consistency in haplotype composition among profiles of identical samples that differed only in the sequencing orientation (i.e. 3ʹ to 5ʹ end or 5ʹ to 3ʹ end) and retained only those haplotypes that were consistently present in both duplicates. After these steps, haplotypes were clustered to Operational Taxonomic Units (OTUs) using *vsearch*^45^ assuming 97% sequence similarity threshold. Taxonomic assignation of OTUs was conducted by *RDP classifier* (80% confidence threshold)^46^ and Silva reference database (v. 132)^47^ (bacterial data) or UNITE database (fungal data).^44^ In specific cases, we applied phylogenetic placement analyses to achieve more detailed OTU assignation. To do so, we extracted all reference 16S rRNA sequences corresponding to the same genus as OTUs in question from the Silva database and clustered them at 99% similarity using *vsearch*.^45^ Representative sequences for clusters exhibiting >97% sequence similarity with any OTU in question were used for phylogenetic reconstruction, which was done by *RAxML*,^48^ assuming the GTRI substitution model after *mafft* alignment.^49^ Bootstrap analysis (1,000 replicates) was conducted to assess the robustness of phylogenetic clades. OTU table, OTU representative sequences, OTU taxonomy, and sample metadata were merged into a single phyloseq database for later statistical calculations.^50^ Bacterial metagenome functional predictions were conducted using *PICRUSt2* pipeline^51^ using default setup, and predicted metagenomes were categorized into functional pathways.^52^ Their predicted abundances were used in later statistical analyses. Weighted NSTI scores (i.e. an index negatively related to the prediction quality and characterizing similarity between 16S rRNA profiles in question and reference genomes) calculated using *PICRUSt2*, were comparable across the three study groups (ANOVA: F_(2,169)_ = 2.62, *p* = 0.0757; mean = 0.136 for controls, 0.162 for AN1, and 0.146 for AN2).

### Statistical analyses of microbiota data

Overall, the bacterial and fungal community composition of 172 or 156 samples was analyzed, respectively. As sequencing coverage varied between samples, we rarefied resulting OTU tables (rarefaction threshold being equal to minimal sequencing coverage) and used the rarefied datasets for further analyses, if not otherwise stated. The observed number of OTUs, Chao 1 total OTU richness estimates, and Shannon indices were included as response variables for alpha diversity analyses. We compared alpha diversities between the three study groups (i.e. control, AN1, and AN2) using linear mixed effect models (LMM), where individual identity was considered as a random effect. Using linear regression on a sample subset corresponding to AN1, we tested for the association between alpha diversity and BMI, EDE-Q scores, or disease length. We also checked if individual-level alpha diversity changes before vs. after hospitalization corresponded to changes in BMI, EDE-Q scores, or disease outcome while accounting for the hospitalization length.

Visual insight into microbiota composition was provided by bar plots for dominating microbiota classes and by Krona hierarchical piecharts.^53^ We also employed Principal Coordinate Analysis (PCoA) running on abundance-based (i.e. Bray-Curtis) and prevalence-based (i.e. binary Jaccard) dissimilarities. Systematic differences in composition and interindividual variation between groups were tested by pair-wise PERMANOVA and betadisper using R package metagMisc. Distance-based redundancy analysis (db-RDA) was applied to test for the association between bacteriome composition and BMI, EDE-Q scores, hyperactivity, or disease length in patients prior to hospitalization. Using linear regression, we also tested if changes in bacteriome composition (expressed as Jaccard and Bray-Curtis dissimilarities) prior vs. after hospitalization correlated with changes in BMI, EDE-Q scores, or “outcome” as well as with the hospital stay length. OTUs and genera, whose abundances varied between controls vs. AN1 and between AN1 vs. AN2, were identified using DESeq2 pipeline.^54^ Mixed models assuming negative binomial error distribution (R package glmmTBM) were employed to test for association between abundances of bacterial OTUs or genera (i.e. response variables) and concentrations of SCFAs or neurotransmitters (i.e. model predictors). The effect of these predictors was statistically controlled for putative variation of OTU/genera abundances among study groups. The individual identity included a random effect and per sample sequencing depth (log scaled) as a model offset. False discovery rates were used to account for false-positive outcomes due to multiple testing.^55^ Due to convergence problems, we fitted this model only for OTUs/genera detected in <10% samples. The same approach was applied to detect associations between biochemical and anthropometric parameters and bacterial OTUs/genera.

For each experimental group, we identified bacterial OTUs that were present in >90% of the sample (hereafter “core” microbiota), and compared variation in the percentage of reads corresponding to these OTUs using analysis of variance (ANOVA).

Variation in predicted proportions of the functional pathway between study groups was analyzed using pair-wise PERMANOVA and betadisper as well as by PCoA. Functional pathways whose abundances varied among study groups were identified using DESeq2 as already described. All statistical analyses were run using R software (version 3.4.4).

### NMR and MS samples preparation

For nuclear magnetic resonance (NMR) analysis, approximately 100 mg aliquot of stool was mixed with water (1 mL, LC-MS grade) and vortexed for 2 min. The homogenized mixture was centrifuged (10 minutes, 14000 rpm, at 4°C). The supernatant was transferred by needle (100 Sterican, 1.20 × 40 mm, Braun, Germany) into a syringe (Omnifix® LuerLockSolo, Germany) and filtered by syringe filter (pore size 0.2 µm, diameter 25 mm, Whatman, UK); the filter was then rinsed with water (0.5 mL). The filtrate was mixed with a methanol/dichloromethane mixture (2:1 v/v, 1 mL), vortexed for 2 min, and centrifuged (30 minutes, 14000 rpm, at 4°C). The upper hydrophilic phase was collected to a fresh vial, evaporated using Speedvac, and stored at −80°C.

Before analysis, dried stool extract samples were dissolved in D_2_O (450 µL), mixed with phosphate buffer (50 µL, 1.5 M KH_2_PO_4_ in D_2_O containing 2 mM NaN_3_ and 0.1% (w/v) trimethylsilyl propionic acid (TSP), pH 7.4), and transferred to 5 mm NMR tubes.

For the mass-spectrometry (MS-based neurotransmitter analysis), an internal standard methionine-d3 (EZ:faast kit, Phenomenex) was added into the next 100-mg stool aliquot. Then, water (1 mL, LC-MS grade) was added, vortexed, centrifuged (10 minutes, 14000 rpm, at 4°C), and filtered in the same way as for the NMR sample preparation. The filtrate (200 µL) was mixed with formic acid (1 µL, MS grade, Honeywell) and derivatized according to the manual (EZ:faast kit, Phenomenex).

### SCFAs analysis

SCFA levels (acetate, butyrate, propionate) in stool samples were assessed by NMR. All NMR experiments were performed at 300 K on a Bruker Avance III 600 MHz spectrometer (Bruker BioSpin, Rheinstetten, Germany) equipped with a 5 mm TCI cryogenic probe head. Nuclear Overhauser effect spectroscopy pulse sequence with presaturation in relaxation delay (1D NOESY, pulse sequence: noesygppr1d) was used for ^1^H NMR experiments (256 scans (NS), 64k of data points (TD), spectral width (SW) of 20 ppm, relaxation delay (d1) of 4s). The free induction decays of the 1D NOESY experiments were multiplied by an exponential window function (LB = 0.3 Hz). The spectra were automatically phased, baseline corrected, and referenced to TSP (0.0 ppm). All spectra were normalized by probabilistic quotient normalization (PQN)^56^ to the group of healthy controls using MATLAB software (MATLAB version 9.2; R2017a). Normalized SCFA concentrations were calculated from methyl signals of acetate (at 1.92 ppm), propionate (at 1.05), and the methylene signal of butyrate (at 1.57 ppm).

### Analysis of neurohormone levels

Derivatives were measured by a mass spectrometer (TSQ Quantum Access Max, Thermo Fisher Scientific, Inc., USA) by the selective reaction monitoring in positive ionization mode. Measurement conditions were optimized by standards (direct infusion of 10 mg/L in the mobile phase, 20 µL/min; Table S4). The injection volume was 10 µL. HESI-II probe was run under the following set up: vaporizer temperature 320°C, spray voltage +2250 V, sheath gas pressure 34.0 AU, auxiliary gas pressure 15.0 AU, ion sweep gas pressure 11.2 AU, collision gas Ar pressure 1.0 mTorr, capillary temperature 320°C. Data were processed by ThernoXcalibur software (Thermo Fisher Scientific, Inc., USA). The peak areas were normalized by the stool sample weight and internal standard area. In stool samples, the adrenaline concentrations were under the detection limit.

## Supplementary Material

Supplemental Material

## Data Availability

Sequencing data are archived in the European Nucleotide Archive under project PRJEB38930. Accession numbers for each sample are available in Table S5 (www.ebi.ac.uk/ena).

## References

[1] Association AP. Diagnostic and statistical manual of mental disorders, 5th ed. Washington (DC, USA): American Psychiatric Publishing, Inc.; 2013.

[2] Liang D, Leung RK, Guan W, Au WW. Involvement of gut microbiome in human health and disease: brief overview, knowledge gaps and research opportunities. Gut Pathog. 2018;10:3. doi:10.1186/s13099-018-0230-4. 29416567 PMC5785832

[3] Levy M, Kolodziejczyk AA, Thaiss CA, Elinav E. Dysbiosis and the immune system. Nat Rev Immunol. 2017 4;17(4):219–25. doi:10.1038/nri.2017.7. 28260787

[4] Lloyd-Price J, Abu-Ali G, Huttenhower C. The healthy human microbiome. Genome Med. 2016 4 27;8(1):51. doi:10.1186/s13073-016-0307-y. 27122046 PMC4848870

[5] Nash AK, Auchtung TA, Wong MC, Smith DP, Gesell JR, Ross MC, Stewart CJ, Metcalf GA, Muzny DM, Gibbs RA, et al. The gut mycobiome of the human microbiome project healthy cohort. Microbiome. 2017 11 25;5(1):153. doi:10.1186/s40168-017-0373-4. 29178920 PMC5702186

[6] Chong CYL, Bloomfield FH, O’Sullivan JM. Factors affecting gastrointestinal microbiome development in neonates. Nutrients. 2018 2 28;10(3):274. doi:10.3390/nu10030274. PMC5872692 29495552

[7] Kleiman SC, Watson HJ, Bulik-Sullivan EC, Huh EY, Tarantino LM, Bulik CM, Carroll IM. The intestinal microbiota in acute anorexia nervosa and during renourishment: relationship to depression, anxiety, and eating disorder psychopathology. Psychosom Med. 2015 Nov-Dec;77(9):969–981. doi:10.1097/PSY.0000000000000247. 26428446 PMC4643361

[8] Armougom F, Henry M, Vialettes B, Raccah D, Raoult D, Ratner AJ. Monitoring bacterial community of human gut microbiota reveals an increase in *Lactobacillus* in obese patients and Methanogens in anorexic patients. PLoS One. 2009 9 23;4(9):e7125. doi:10.1371/journal.pone.0007125. 19774074 PMC2742902

[9] Morita C, Tsuji H, Hata T, Gondo M, Takakura S, Kawai K, Yoshihara K, Ogata K, Nomoto K, Miyazaki K, et al. Gut dysbiosis in patients with anorexia nervosa. PLoS One. 2015;10(12):e0145274. doi:10.1371/journal.pone.0145274. 26682545 PMC4687631

[10] Million M, Angelakis E, Maraninchi M, Henry M, Giorgi R, Valero R, Vialettes B, Raoult D. Correlation between body mass index and gut concentrations of *Lactobacillus reuteri, Bifidobacterium animalis, Methanobrevibacter smithii* and *Escherichia coli*. Int J Obes (Lond). 2013 11;37(11):1460–1466. doi:10.1038/ijo.2013.20. 23459324 PMC3826031

[11] Borgo F, Riva A, Benetti A, Casiraghi MC, Bertelli S, Garbossa S, Anselmetti S, Scarone S, Pontiroli AE, Morace G, et al. Microbiota in anorexia nervosa: the triangle between bacterial species, metabolites and psychological tests. PLoS One. 2017;12(6):e0179739. doi:10.1371/journal.pone.0179739. 28636668 PMC5479564

[12] Morkl S, Lackner S, Muller W, Gorkiewicz G, Kashofer K, Oberascher A, Painold A, Holl A, Holzer P, Meinitzer A, et al. Gut microbiota and body composition in anorexia nervosa inpatients in comparison to athletes, overweight, obese, and normal weight controls. Int J Eat Disord. 2017 12;50(12):1421–1431. doi:10.1002/eat.22801. 29131365

[13] Mack I, Cuntz U, Gramer C, Niedermaier S, Pohl C, Schwiertz A, Zimmermann K, Zipfel S, Enck P, Penders J. Weight gain in anorexia nervosa does not ameliorate the faecal microbiota, branched chain fatty acid profiles, and gastrointestinal complaints. Sci Rep. 2016 5;27(6):26752. doi:10.1038/srep26752. PMC4882621 27229737

[14] Hanachi M, Manichanh C, Schoenenberger A, Pascal V, Levenez F, Cournede N, Dore J, Melchior JC. Altered host-gut microbes symbiosis in severely malnourished anorexia nervosa (AN) patients undergoing enteral nutrition: an explicative factor of functional intestinal disorders? Clin Nutr. 2019 10;38(5):2304–2310. doi:10.1016/j.clnu.2018.10.004. 30527539

[15] Monteleone AM, Troisi J, Fasano A, Dalle Grave R, Marciello F, Serena G, Calugi S, Scala G, Corrivetti G, Cascino G, et al. Multi-omics data integration in anorexia nervosa patients before and after weight regain: a microbiome-metabolomics investigation. Clin Nutr. 2020 7 31;40(3):1137–1146. doi:10.1016/j.clnu.2020.07.021. 32782162

[16] Neuman H, Debelius JW, Knight R, Koren O. Microbial endocrinology: the interplay between the microbiota and the endocrine system. FEMS Microbiol Rev. 2015 7;39(4):509–521. doi:10.1093/femsre/fuu010. 25701044

[17] Nicholson JK, Holmes E, Kinross J, Burcelin R, Gibson G, Jia W, Pettersson S. Host-gut microbiota metabolic interactions. Science. 2012 6 8;336(6086):1262–1267. doi:10.1126/science.1223813. 22674330

[18] Wong JM, De Souza R, Kendall CW, Emam A, Jenkins DJ. Colonic health: fermentation and short chain fatty acids. J Clin Gastroenterol. 2006 3;40(3):235–243. doi:10.1097/00004836-200603000-00015. 16633129

[19] Carabotti M, Scirocco A, Maselli MA, Severi C. The gut-brain axis: interactions between enteric microbiota, central and enteric nervous systems. Ann Gastroenterol. 2015 Apr-Jun;28(2):203–209. 25830558 PMC4367209

[20] Roubalova R, Prochazkova P, Papezova H, Smitka K, Bilej M, Tlaskalova-Hogenova H. Anorexia nervosa: gut microbiota-immune-brain interactions. Clin Nutr. 2020 3;39(3):676–684. doi:10.1016/j.clnu.2019.03.023. 30952533

[21] Shetty SA, Hugenholtz F, Lahti L, Smidt H, De Vos WM. Intestinal microbiome landscaping: insight in community assemblage and implications for microbial modulation strategies. FEMS Microbiol Rev. 2017 3 1;41(2):182–199. doi:10.1093/femsre/fuw045. 28364729 PMC5399919

[22] Furtado M, Katzman MA. Neuroinflammatory pathways in anxiety, posttraumatic stress, and obsessive compulsive disorders. Psychiatry Res. 2015 9 30;229(1–2):37–48. doi:10.1016/j.psychres.2015.05.036. 26296951

[23] Wales J, Brewin N, Cashmore R, Haycraft E, Baggott J, Cooper A, Arcelus J. Predictors of positive treatment outcome in people with anorexia nervosa treated in a specialized inpatient unit: the role of early response to treatment. Eur Eat Disord Rev. 2016 9;24(5):417–424. doi:10.1002/erv.2443. 27045727

[24] Zaneveld JR, McMinds R, Vega Thurber R. Stress and stability: applying the Anna Karenina principle to animal microbiomes. Nat Microbiol. 2017 8;24(2):17121. doi:10.1038/nmicrobiol.2017.121. 28836573

[25] Parker BJ, Wearsch PA, Veloo ACM, Rodriguez-Palacios A. The genus *Alistipes*: gut bacteria with emerging implications to inflammation, cancer, and mental health. Front Immunol. 2020;11. doi:10.3389/fimmu.2020.00906. PMC7296073 32582143

[26] Jiang H, Ling Z, Zhang Y, Mao H, Ma Z, Yin Y, Wang W, Tang W, Tan Z, Shi J, et al. Altered fecal microbiota composition in patients with major depressive disorder. Brain Behav Immun. 2015 8;48:186–194. doi:10.1016/j.bbi.2015.03.016. 25882912

[27] Goodrich JK, Waters JL, Poole AC, Sutter JL, Koren O, Blekhman R, Beaumont M, Van Treuren W, Knight R, Bell JT, et al. Human genetics shape the gut microbiome. Cell. 2014 11 6;159(4):789–799. doi:10.1016/j.cell.2014.09.053. 25417156 PMC4255478

[28] Ruppin H, Bar-Meir S, Soergel KH, Wood CM, Schmitt MG Jr. Absorption of short-chain fatty acids by the colon. Gastroenterology. 1980 6;78(6):1500–1507. doi:10.1016/S0016-5085(19)30508-6. 6768637

[29] Gao J, Cahill CM, Huang X, Roffman JL, Lamon-Fava S, Fava M, Mischoulon D, Rogers JT. S-adenosyl methionine and transmethylation pathways in neuropsychiatric diseases throughout life. Neurotherapeutics. 2018 1;15(1):156–175. 29340929 10.1007/s13311-017-0593-0 PMC5794704

[30] De Berardis D, Orsolini L, Serroni N, Girinelli G, Iasevoli F, Tomasetti C, De Bartolomeis A, Mazza M, Valchera A, Fornaro M, et al. A comprehensive review on the efficacy of S-adenosyl-L-methionine in major depressive disorder. CNS Neurol Disord Drug Targets. 2016;15(1):35–44. doi:10.2174/1871527314666150821103825. 26295824

[31] Hoffmann C, Dollive S, Grunberg S, Chen J, Li H, Wu GD, Lewis JD, Bushman FD. Archaea and fungi of the human gut microbiome: correlations with diet and bacterial residents. PLoS One. 2013;8(6):e66019. doi:10.1371/journal.pone.0066019. 23799070 PMC3684604

[32] Gabaldon T, Martin T, Marcet-Houben M, Durrens P, Bolotin-Fukuhara M, Lespinet O, Arnaise S, Boisnard S, Aguileta G, Atanasova R, et al. Comparative genomics of emerging pathogens in the *Candida glabrata* clade. BMC Genomics. 2013 9;14(14):623. doi:10.1186/1471-2164-14-623. 24034898 PMC3847288

[33] Prochazkova P, Roubalova R, Dvorak J, Tlaskalova-Hogenova H, Cermakova M, Tomasova P, Sediva B, Kuzma M, Bulant J, Bilej M, et al. Microbiota, microbial metabolites, and barrier function in a patient with anorexia nervosa after fecal microbiota transplantation. Microorganisms. 2019 9 10;7(9):338. doi:10.3390/microorganisms7090338. PMC6780752 31510101

[34] Kaye WH, Fudge JL, Paulus M. New insights into symptoms and neurocircuit function of anorexia nervosa. Nat Rev Neurosci. 2009 8;10(8):573–584. doi:10.1038/nrn2682. 19603056 PMC13038070

[35] Cryan JF, Kaupmann K. Don’t worry ‘B’ happy!: a role for GABA(B) receptors in anxiety and depression. Trends Pharmacol Sci. 2005 1;26(1):36–43. doi:10.1016/j.tips.2004.11.004. 15629203

[36] Den Besten G, Van Eunen K, Groen AK, Venema K, Reijngoud DJ, Bakker BM. The role of short-chain fatty acids in the interplay between diet, gut microbiota, and host energy metabolism. J Lipid Res. 2013 9;54(9):2325–2340. doi:10.1194/jlr.R036012. 23821742 PMC3735932

[37] Fairburn CG. Eating disorders: the transdiagnostic view and the cognitive behavioral theory. In: Fairburn CG, editor. Cognitive behavior therapy and eating disorders. New York, USA: Guilford Press; 2008. p. 7–22.

[38] Fairburn CG, Beglin SJ. Assessment of eating disorders: interview or self-report questionnaire? Int J Eat Disord. 1994 12;16(4):363–370. 7866415

[39] Caporaso JG, Lauber CL, Walters WA, Berg-Lyons D, Lozupone CA, Turnbaugh PJ, Fierer N, Knight R. Global patterns of 16S rRNA diversity at a depth of millions of sequences per sample. Proc Natl Acad Sci U S A. 2011 3 15;108(Suppl 1):4516–4522. doi:10.1073/pnas.1000080107. 20534432 PMC3063599

[40] Ihrmark K, Bodeker IT, Cruz-Martinez K, Friberg H, Kubartova A, Schenck J, Strid Y, Stenlid J, Brandstrom-Durling M, Clemmensen KE, et al. New primers to amplify the fungal ITS2 region–evaluation by 454-sequencing of artificial and natural communities. FEMS Microbiol Ecol. 2012 12;82(3):666–677. doi:10.1111/j.1574-6941.2012.01437.x. 22738186

[41] Jiang H, Lei R, Ding SW, Zhu S. Skewer: a fast and accurate adapter trimmer for next-generation sequencing paired-end reads. BMC Bioinform. 2014 6;12(15):182. doi:10.1186/1471-2105-15-182. PMC4074385 24925680

[42] Callahan BJ, McMurdie PJ, Rosen MJ, Han AW, Johnson AJ, Holmes SP. DADA2: high-resolution sample inference from Illumina amplicon data. Nat Methods. 2016 7;13(7):581–583. doi:10.1038/nmeth.3869. 27214047 PMC4927377

[43] Edgar RC, Haas BJ, Clemente JC, Quince C, Knight R. UCHIME improves sensitivity and speed of chimera detection. Bioinformatics. 2011 8 15;27(16):2194–2200. doi:10.1093/bioinformatics/btr381. 21700674 PMC3150044

[44] Nilsson RH, Larsson KH, Taylor AFS, Bengtsson-Palme J, Jeppesen TS, Schigel D, Kennedy P, Picard K, Glockner FO, Tedersoo L, et al. The UNITE database for molecular identification of fungi: handling dark taxa and parallel taxonomic classifications. Nucleic Acids Res. 2019 1 8;47(D1):D259–D264. doi:10.1093/nar/gky1022. 30371820 PMC6324048

[45] Rognes T, Flouri T, Nichols B, Quince C, Mahe F. VSEARCH: a versatile open source tool for metagenomics. PeerJ. 2016;4:e2584. doi:10.7717/peerj.2584. 27781170 PMC5075697

[46] Wang Q, Garrity GM, Tiedje JM, Cole JR. Naive Bayesian classifier for rapid assignment of rRNA sequences into the new bacterial taxonomy. Appl Environ Microbiol. 2007 8;73(16):5261–5267. doi:10.1128/AEM.00062-07. 17586664 PMC1950982

[47] Quast C, Pruesse E, Yilmaz P, Gerken J, Schweer T, Yarza P, Peplies J, Glockner FO. The SILVA ribosomal RNA gene database project: improved data processing and web-based tools. Nucleic Acids Res. 2013 1;41(D1):D590–6. doi:10.1093/nar/gks1219. 23193283 PMC3531112

[48] Stamatakis A. RAxML version 8: a tool for phylogenetic analysis and post-analysis of large phylogenies. Bioinformatics. 2014 5 1;30(9):1312–1313. doi:10.1093/bioinformatics/btu033. 24451623 PMC3998144

[49] Katoh K, Standley DM. MAFFT multiple sequence alignment software version 7: improvements in performance and usability. Mol Biol Evol. 2013 4;30(4):772–780. doi:10.1093/molbev/mst010. 23329690 PMC3603318

[50] McMurdie PJ, Holmes S, Watson M. phyloseq: an R package for reproducible interactive analysis and graphics of microbiome census data. PLoS One. 2013;8(4):e61217. doi:10.1371/journal.pone.0061217. 23630581 PMC3632530

[51] Douglas GM, Maffei VJ, Zanevel J, Yurgel SN, Brown JR, Christopher M, Taylor CM, Huttenhower C, Langille MGI. PICRUSt2: an improved and customizable approach for metagenome inference. BioRxiv. 2020. doi:10.1101/672295

[52] Ye Y, Doak TG, Ouzounis CA. A parsimony approach to biological pathway reconstruction/inference for genomes and metagenomes. PLoS Comput Biol. 2009 8;5(8):e1000465. doi:10.1371/journal.pcbi.1000465. 19680427 PMC2714467

[53] Ondov BD, Bergman NH, Phillippy AM. Interactive metagenomic visualization in a Web browser. BMC Bioinform. 2011 9;30(12):385. doi:10.1186/1471-2105-12-385. PMC3190407 21961884

[54] Love MI, Huber W, Anders S. Moderated estimation of fold change and dispersion for RNA-seq data with DESeq2. Genome Biol. 2014;15(12):550. doi:10.1186/s13059-014-0550-8. 25516281 PMC4302049

[55] Benjamini Y, Hochberg Y. Controlling the false discovery rate - a practical and powerful approach to multiple testing. J R Stat Soc Ser B-Stat Methodol. 1995;57:289–300.

[56] Dieterle F, Ross A, Schlotterbeck G, Senn H. Probabilistic quotient normalization as robust method to account for dilution of complex biological mixtures. Application in 1H NMR metabonomics. Anal Chem. 2006 7 1;78(13):4281–4290. doi:10.1021/ac051632c. 16808434

