# Supplementary material for: The intestinal microbiota and metabolites in patients with anorexia nervosa
Source: Gut Microbes. 2021 Mar 28;13(1):1902771. doi: 10.1080/19490976.2021.1902771 (PMC8018350; doi:10.1080/19490976.2021.1902771)
Supplement: Supplemental Material [file KGMI_A_1902771_SM3349.zip › Prochazkova_gut_microbes_supplements_revision3_final.docx]

**Supplemental Material**

**Supplemental tables**

Table S1. EDE-Q subscales correlations.

| A |  |  |  |  |  |  |  | B |  |  |  |  |
| --- | --- | --- | --- | --- | --- | --- | --- | --- | --- | --- | --- | --- |
|  | | EDE-Q restraint concern | EDE-Q eating concern | EDE-Q shape concern | EDE-Q weight concern | EDE-Q total score |  | EDE-Q restraint concern | EDE-Q eating concern | EDE-Q shape concern | EDE-Q weight concern | EDE-Q total score |
| EDE-Q restraint concern | *r* |  | 0.699 | 0.680 | 0.545 | 0.852 |  |  | 0.552 | 0.436 | 0.495 | 0.793 |
|  | *p* |  | 0.000 | 0.000 | 0.000 | 0.000 |  |  | 0.000 | 0.002 | 0.000 | 0.000 |
| EDE-Q eating concern | *r* | 0.699 |  | 0.740 | 0.771 | 0.902 |  | 0.552 |  | 0.452 | 0.651 | 0.796 |
|  | *p* | 0.000 |  | 0.000 | 0.000 | 0.000 |  | 0.000 |  | 0.001 | 0.000 | 0.000 |
| EDE-Q shape concern | *r* | 0.680 | 0.740 |  | 0.852 | 0.906 |  | 0.436 | 0.452 |  | 0.813 | 0.805 |
|  | *p* | 0.000 | 0.000 |  | 0.000 | 0.000 |  | 0.002 | 0.001 |  | 0.000 | 0.000 |
| EDE-Q weight concern | *r* | 0.545 | 0.771 | 0.852 |  | 0.873 |  | 0.495 | 0.651 | 0.813 |  | 0.883 |
|  | *p* | 0.000 | 0.000 | 0.000 |  | 0.000 |  | 0.000 | 0.000 | 0.000 |  | 0.000 |
| EDE-Q total score | *r* | 0.852 | 0.902 | 0.906 | 0.873 |  |  | 0.793 | 0.796 | 0.805 | 0.883 |  |
|  | *p* | 0.000 | 0.000 | 0.000 | 0.000 |  |  | 0.000 | 0.000 | 0.000 | 0.000 |  |

Pearson's correlations of individual EDE-Q scores of A) patients with AN prior to hospitalization (n=59), B) after the hospitalization (n=52)

Cronbachs Alpha = 0.819 Cronbachs Alpha = 0.893

Standardized Cronbachs Alpha = 0.947 Standardized Cronbachs Alpha = 0.909

Table S2. Relationships between outcome and predictors for the 1^st^ predictive component as evaluated by the OPLS model and MR.

|  |  | **Predictive component** | |  |  |  | **Multiple regression** | |  |
| --- | --- | --- | --- | --- | --- | --- | --- | --- | --- |
|  | **Variable** | Component loading | t-statistics | R*^a^* |  |  | Regression coefficient | t-statistics |  |
| Relevant predictors (matrix **X**) | Hospitalization duration (days) | -0.241 | -5.48 | -0.538 | ** |  | -0.071 | -3.86 | ** |
|  | Disease duration (months) | 0.241 | 7.81 | 0.538 | ** |  | 0.073 | 4.35 | ** |
|  | Albumin (g/l) | -0.136 | -2.17 | -0.303 | * |  | -0.056 | -2.27 | * |
|  | Kynurenine (PQN) | -0.174 | -2.67 | -0.371 | * |  | -0.061 | -3.21 | ** |
|  | Acetate (PQN) | 0.124 | 2.63 | 0.268 | * |  | 0.041 | 2.07 | * |
|  | Adulthood stress | 0.227 | 3.38 | 0.506 | ** |  | 0.079 | 2.94 | * |
|  | No somatic diagnosis | -0.234 | -6.62 | -0.521 | ** |  | -0.081 | -3.45 | ** |
|  | Disability pension | -0.192 | 4.90 | 0.655 | ** |  | 0.103 | 3.80 | ** |
|  | University education | -0.065 | -1.36 | -0.145 |  |  | -0.038 | -3.83 | ** |
|  | Second education | -0.081 | -1.45 | -0.180 |  |  | -0.035 | -3.98 | ** |
|  | Basic education | 0.009 | 0.19 | 0.021 |  |  | -0.026 | -2.32 | * |
|  | No medication | -0.254 | -6.38 | -0.567 | ** |  | -0.066 | -7.04 | ** |
|  | Antidepressants | 0.167 | 3.04 | 0.372 | ** |  | 0.067 | 3.20 | ** |
|  | Other medication | 0.262 | 7.61 | 0.585 | ** |  | 0.091 | 4.33 | ** |
|  | ∆BMI (kg/m^2^) | -0.287 | -7.31 | -0.639 | ** |  | -0.076 | -4.40 | ** |
|  | ∆Body Fat (%) | -0.305 | -7.93 | -0.710 | ** |  | -0.105 | -5.19 | ** |
|  | ∆Waist (cm) | -0.235 | -4.79 | -0.532 | ** |  | -0.062 | -3.24 | ** |
|  | ∆Hip (cm) | -0.235 | -4.75 | -0.573 | ** |  | -0.063 | -2.76 | * |
|  | ∆Albumin (g/l) | 0.155 | 4.30 | 0.346 | ** |  | 0.045 | 4.85 | ** |
|  | ∆AN DSM mild | -0.244 | -4.43 | -0.544 | ** |  | -0.052 | -2.55 | * |
|  | ∆antidepressants | -0.051 | -1.09 | -0.114 |  |  | -0.022 | -3.09 | ** |
| (matrix **Y**) | Outcome 0_LRR | 1.000 | 16.29 | 0.734 | ** |  |  |  |  |
| **Explained variability** | | 53.9% (47.9% after cross-validation) | | | | | | |  |

^a^R Component loadings expressed as correlation coefficients with predictive component, *p<0.05, **p<0.01

^b^LRR Logarithm of likelihood ratio (logarithm of the ratio of the probability that the patient's psychopathology improved to the probability that not); PQN–probabilistic quotient normalization

Table S3. The original microbial community standards' compositions (ZymoBIOMICS^TM^) compared to obtained sequencing data.

| **Microbial community standard** | **gDNA %** | **16S %** | **obtained 16S %** |
| --- | --- | --- | --- |
| *Pseudomonas aeruginosa* | 12 | 4.2 | 7.57 |
| *Escherichia coli* | 12 | 10.1 | 14.61 |
| *Salmonella enterica* | 12 | 10.4 | 13.27 |
| *Lactobacillus fermentum* | 12 | 18.4 | 14.41 |
| *Enterococcus faecalis* | 12 | 9.9 | 7.79 |
| *Staphylococcus aureus* | 12 | 15.5 | 12.43 |
| *Listeria monocytogenes* | 12 | 14.1 | 13.75 |
| *Bacillus subtilis* | 12 | 17.4 | 16.18 |
| *Saccharomyces cerevisiae* | 2 | na | na |
| *Cryptococcus neoformans* | 2 | na | na |

| **Microbial community standard II** | **gDNA %** | **16S %** | **obtained 16S %** |
| --- | --- | --- | --- |
| *Listeria monocytogenes* | 89.1 | 95.9 | 81.11 |
| *Pseudomonas aeruginosa* | 8.9 | 2.8 | 16.11 |
| *Bacillus subtilis* | 0.89 | 1.2 | 1.69 |
| *Saccharomyces cerevisiae* | 0.89 | na | na |
| *Escherichia coli* | 0.089 | 0.069 | 0.57 |
| *Salmonella enterica* | 0.089 | 0.07 | 0.50 |
| *Lactobacillus fermentum* | 0.0089 | 0.012 | 0.03 |
| *Enterococcus faecalis* | 0.0089 | 0.00067 | nd |
| *Cryptococcus neoformans* | 0.00089 | na | na |
| *Staphylococcus aureus* | 0.00089 | 0.0001 | nd |

| **Microbial community DNA standard** | **gDNA %** | **16S %** | **obtained 16S %** |
| --- | --- | --- | --- |
| *Pseudomonas aeruginosa* | 12 | 4.2 | 9.78 |
| *Escherichia coli* | 12 | 10.1 | 13.02 |
| *Salmonella enterica* | 12 | 10.4 | 11.05 |
| *Lactobacillus fermentum* | 12 | 18.4 | 15.05 |
| *Enterococcus faecalis* | 12 | 9.9 | 7.97 |
| *Staphylococcus aureus* | 12 | 15.5 | 14.66 |
| *Listeria monocytogenes* | 12 | 14.1 | 12.43 |
| *Bacillus subtilis* | 12 | 17.4 | 16.05 |
| *Saccharomyces cerevisiae* | 2 | na | na |
| *Cryptococcus neoformans* | 2 | na | na |

| **Microbial community DNA standard II** | **gDNA %** | **16S %** | **obtained 16S %** |
| --- | --- | --- | --- |
| *Listeria monocytogenes* | 89.1 | 95.9 | 74.53 |
| *Pseudomonas aeruginosa* | 8.9 | 2.8 | 22.79 |
| *Bacillus subtilis* | 0.89 | 1.2 | 1.62 |
| *Saccharomyces cerevisiae* | 0.89 | na | na |
| *Escherichia coli* | 0.089 | 0.069 | 0.52 |
| *Salmonella enterica* | 0.089 | 0.07 | 0.52 |
| *Lactobacillus fermentum* | 0.0089 | 0.012 | 0.03 |
| *Enterococcus faecalis* | 0.0089 | 0.00067 | nd |
| *Cryptococcus neoformans* | 0.00089 | na | na |
| *Staphylococcus aureus* | 0.00089 | 0.0001 | nd |

na – not assessed; nd – not detected

The theoretical composition in terms of 16S rRNA gene abundance was calculated from theoretical genomic DNA composition with the following formula: 16S copy number = total genomic DNA (g) × unit conversion constant (bp/g) / genome size (bp) × 16S copy number per genome. The obtained 16S% was calculated as an average of abundances from both forward and reverse sequencing raw data.

Table S4. Identification parameters used in MS.

| Metabolite | Parent ion | Production | Collision energy (V) | Tube lens voltage (V) | Retention time (min) |
| --- | --- | --- | --- | --- | --- |
| GABA | 232.0 | 86.112 | 17.18 | 38 | 5.9 |
| Hydroxytryptofan | 435.1 | 201.375 | 29.12 | 69 | 11.6 |
| Tyramine | 310.1 | 121.164 | 28.17 | 72 | 9.9 |
| Adrenalin | 424.0 | 166.252 | 28.19 | 86 | 11.4 |
| Dopamine | 412.0 | 137.180 | 32.20 | 83 | 12.1 |
| Kynurenine | 423.0 | 146.260 | 33.15 | 64 | 15.5 |
| Serotonin | 349.1 | 160.203 | 30.17 | 60 | 8.4 |

**Supplemental figures**


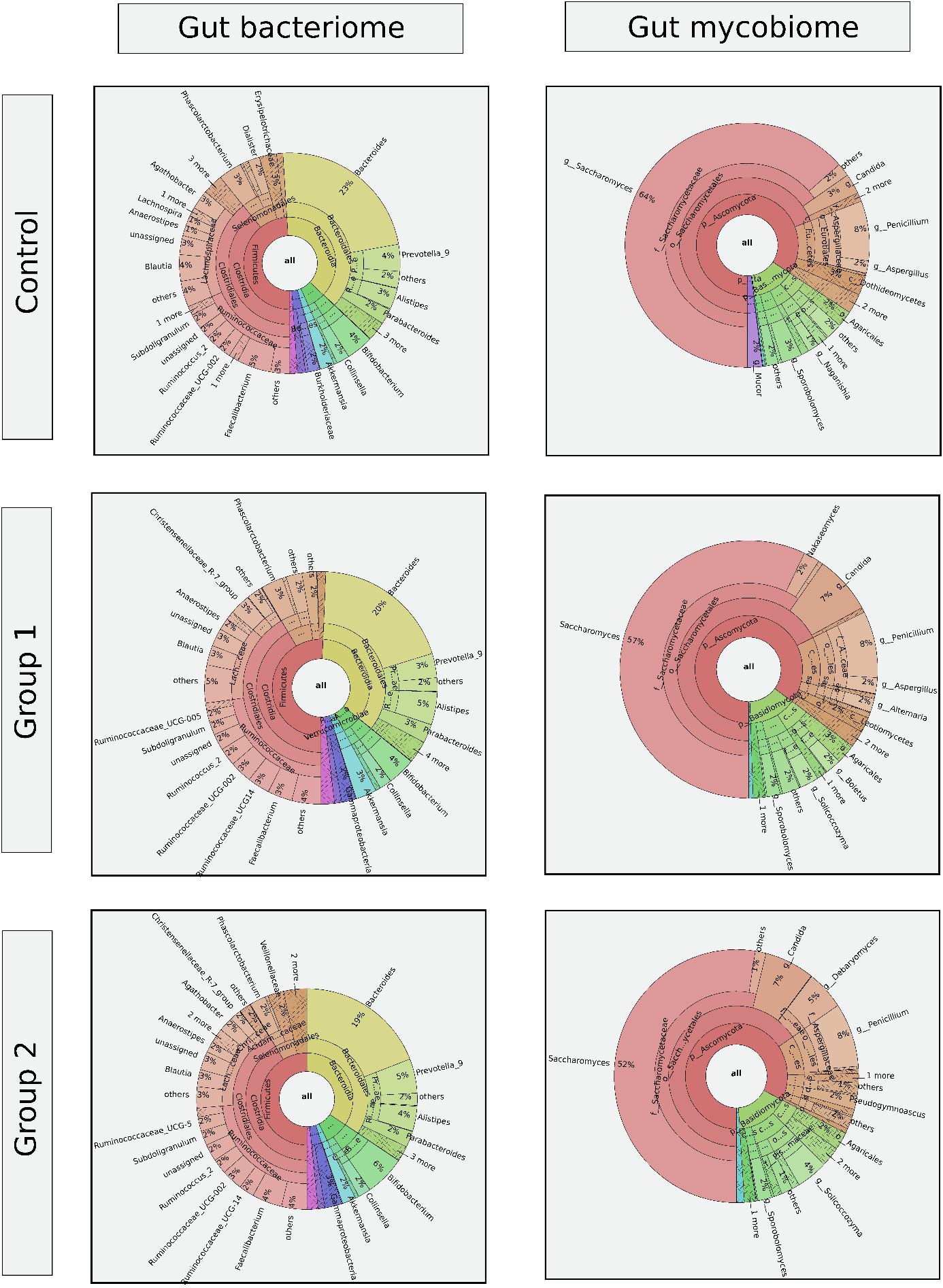


Fig. S1. Average representation of bacterial and fungal taxa in the three studied groups.


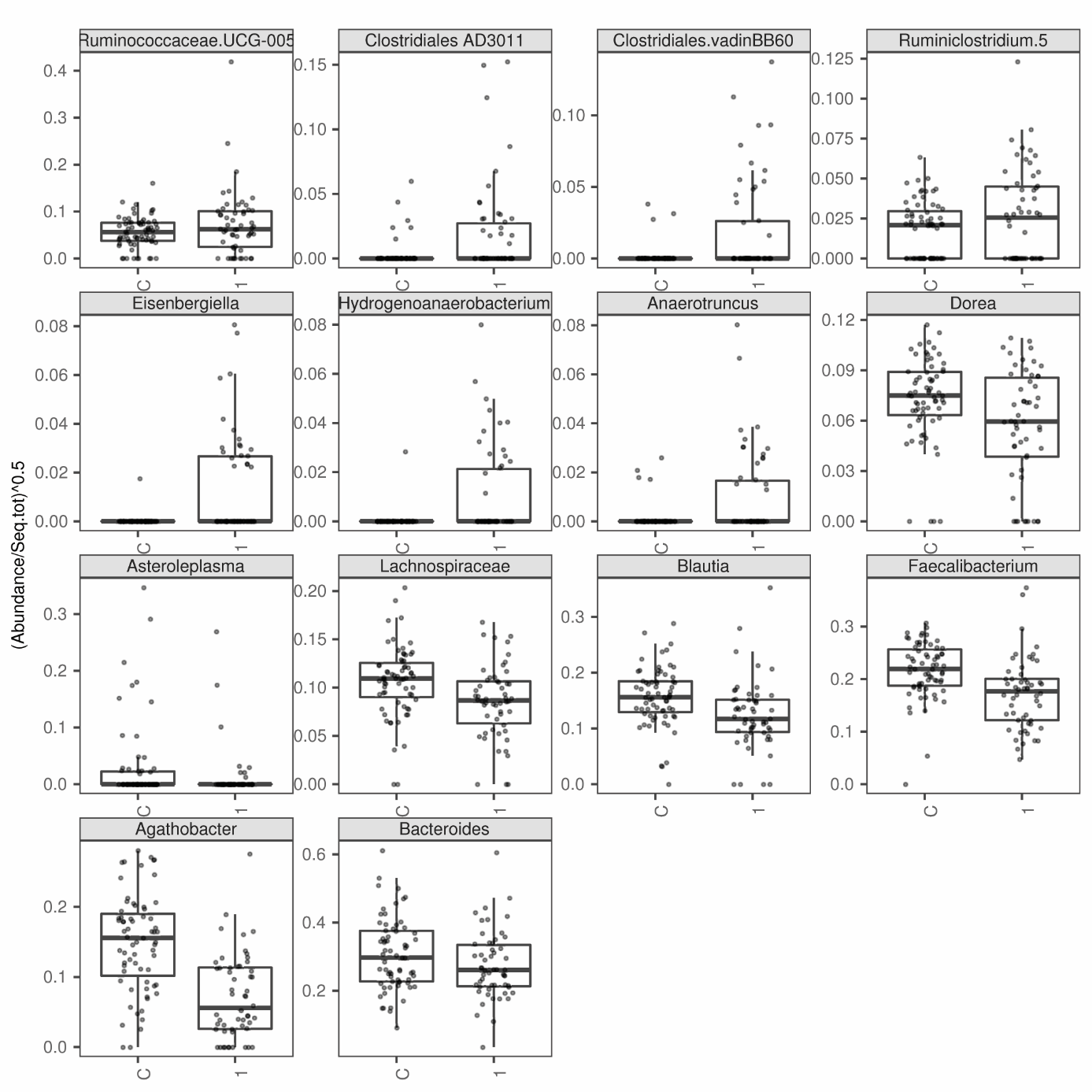


Fig. S2. Relative abundances of bacterial genera (squared-root transformed) that varied. according to DESeq2 analyses (FDR < 0.05) between control samples vs. AN1.


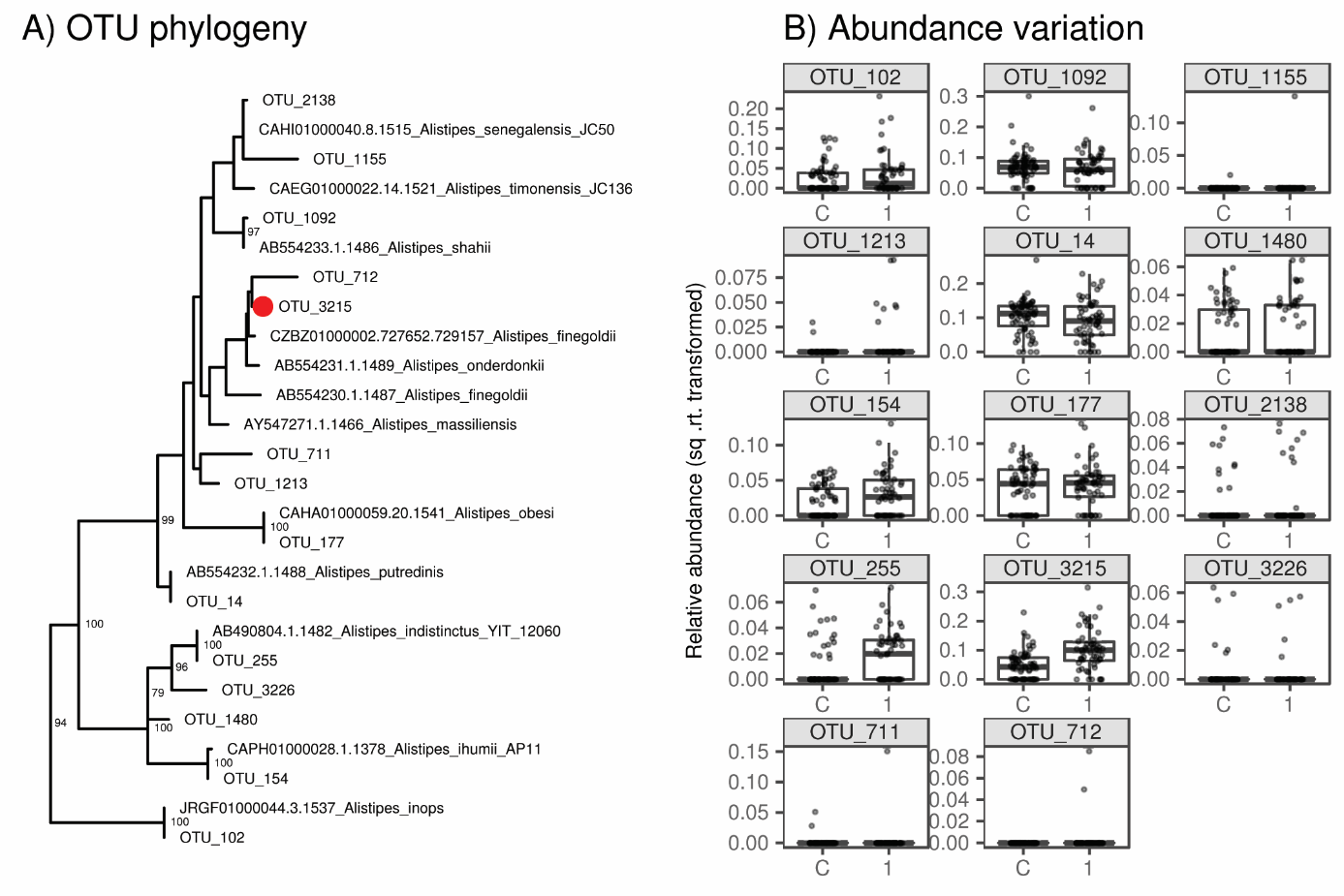


Fig. S3. A) Phylogenetic placement of *Alistipes* OTUs. Bootstrap values > 70 are shown. OTUs exhibiting significant abundance variation between controls vs. AN1 are highlighted. B) Boxplot illustrating an abundance variation of all *Alistipes* OTUs in controls and AN1.


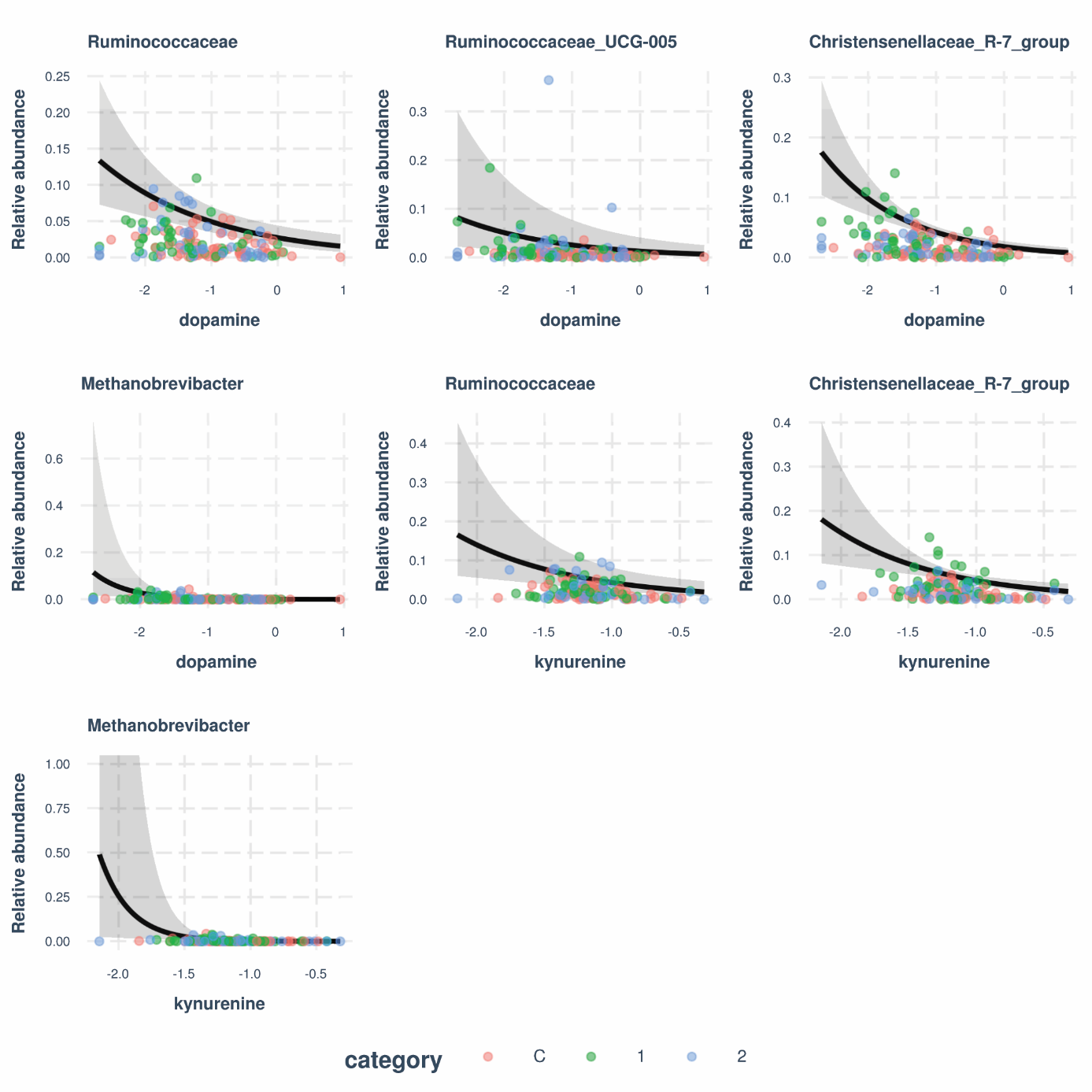


Fig. S4. Significant associations between bacterial genera abundances and concentrations of short-chain fatty acids or neurotransmitters. Predictions and 95% confidence intervals for negative binomial generalized linear mixed models (GLMMs) are shown.


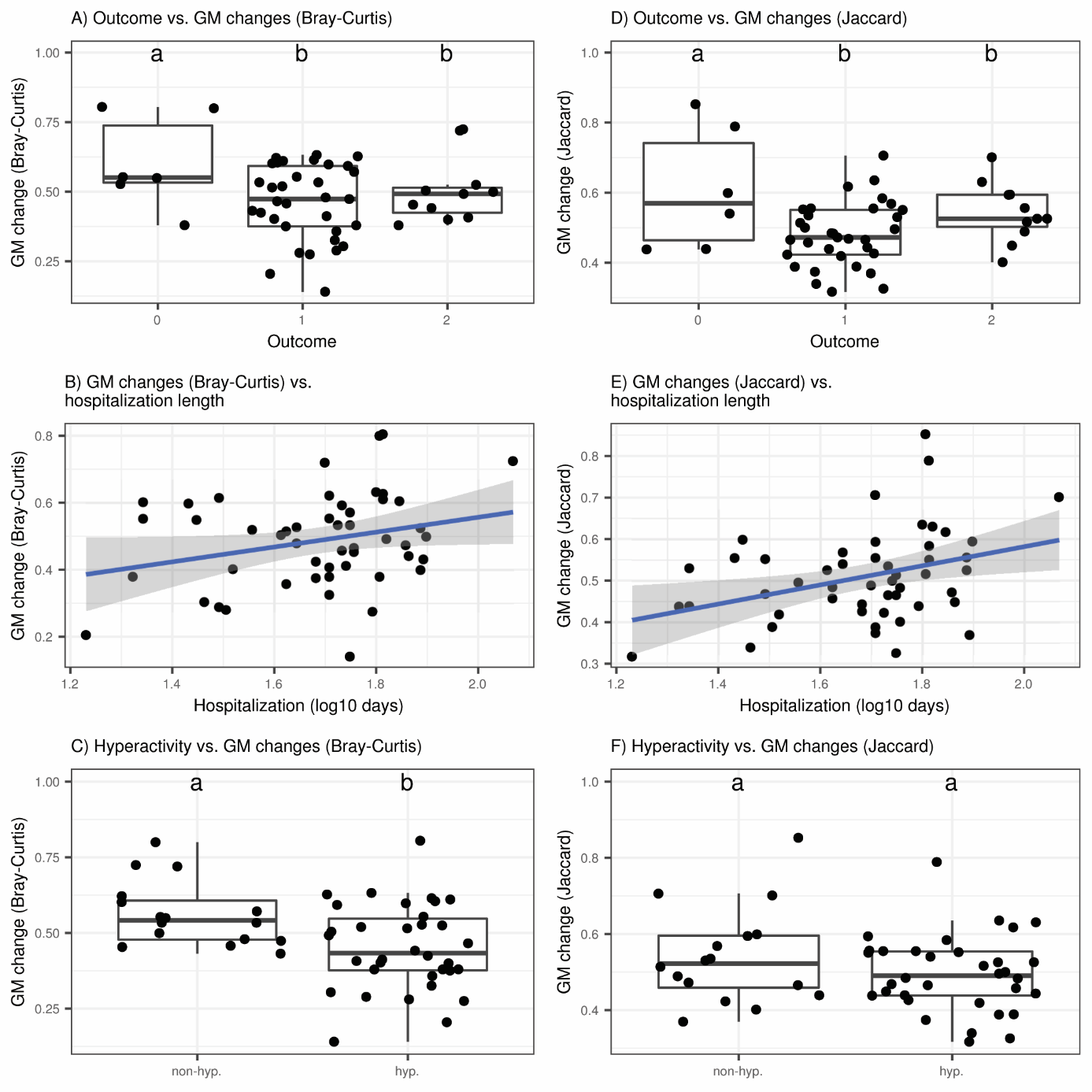


Fig. S5. Association between gut bacteriome changes during hospitalization and A) outcome (Bray-Curtis); D) outcome (Jaccard); B) gut bacteriome changes (Bray-Curtis) and hospitalization length); E) gut bacteriome changes (Jaccard) and hospitalization length (log10 scaled days); C) gut bacteriome changes (Bray-Curtis) and hyperactivity; F) gut bacteriome changes (Jaccard) and hyperactivity. Significant differences among categories (p < 0.05 according to Tukey post-hoc tests) are indicated by different letters.


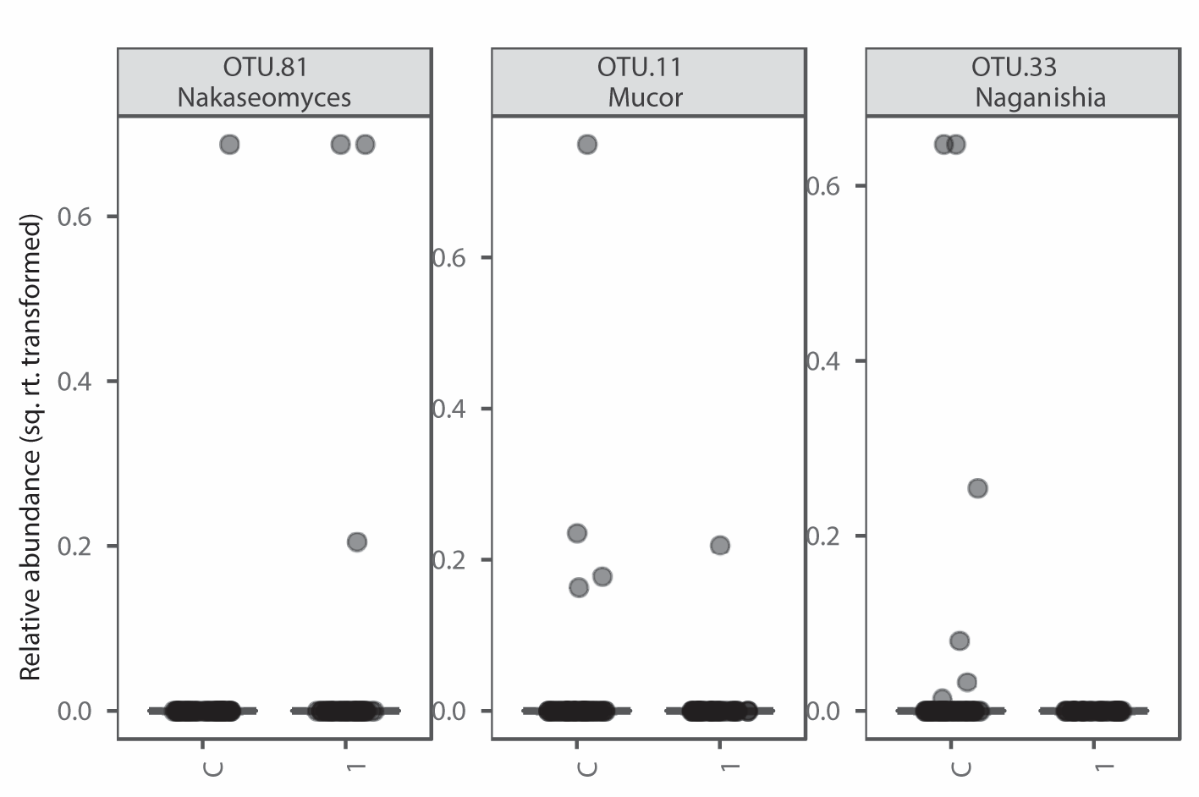


Fig. S6. Relative abundances of fungal OTUs (squared-root transformed) that varied between controls vs. AN1 group according to DESeq2 analyzes.
